# TRPA1s act as chemosensors but not as cold sensors or mechanosensors to trigger the swallowing reflex in rats

**DOI:** 10.1038/s41598-022-07400-3

**Published:** 2022-03-02

**Authors:** Mohammad Zakir Hossain, Hiroshi Ando, Shumpei Unno, Junichi Kitagawa

**Affiliations:** 1 Department of Oral Physiology, School of Dentistry, Matsumoto Dental University, 1780 Gobara Hirooka, Shiojiri, Nagano, 399-0781 Japan; 2 Department of Biology, School of Dentistry, Matsumoto Dental University, Shiojiri, Japan

**Keywords:** Dysphagia, Neurophysiology

## Abstract

We examined the role of TRPA1s in triggering the swallowing reflex. TRPA1s predominantly localized on thin nerve fibers and fibroblast-like cells in swallowing-related regions and on small to medium-sized superior laryngeal nerve-afferents in the nodose–petrosal–jugular ganglionic complex. Topical application of a TRPA1 agonist, allyl isothiocyanate (AITC), dose-dependently triggered swallowing reflexes. Prior topical application of a TRPA1 antagonist significantly attenuated the AITC-induced reflexes. Application of cold AITC (4 °C) very briefly reduced the on-site temperature to < 17 °C (temperature at which TRPA1s can be activated), but had no effect on triggering of the reflex. By contrast, reducing the on-site temperature to < 17 °C for a longer time by continuous flow of cold AITC or by application of iced AITC paradoxically delayed/prevented the triggering of AITC-induced reflexes. Prior application of the TRPA1 antagonist had no effect on the threshold for the punctate mechanical stimuli-induced reflex or the number of low-force or high-force continuous mechanical pressure stimuli-induced reflexes. TRPA1s are functional and act as chemosensors, but not as cold sensors or mechanosensors, for triggering of the swallowing reflex. A brief cold stimulus has no effect on triggering of the reflex. However, a longer cold stimulus delays/prevents triggering of the reflex because of cold anesthesia.

## Introduction

The sensory inputs from the pharyngeal and laryngeal regions play an important role to activate the swallowing central pattern generator (sCPG) located in the brainstem in triggering the swallowing reflex^1–4^. The pharyngeal and associated regions are mainly supplied by a nerve plexus formed by the pharyngeal branches of the glossopharyngeal (IX-ph) and vagus (X-ph) nerves^1,5,6^. The laryngopharyngeal and associated laryngeal regions are mainly supplied by superior laryngeal nerve (SLN), a branch of the vagus nerve^7,8^. The cell bodies of the glossopharyngeal and vagal nerve afferents supplying the swallowing-related regions are located in the nodose–petrosal–jugular ganglionic complex (NPJc)^1–4^. The laryngeal and associated esophageal regions are also supplied by spinal nerves^9^. However, the sensory inputs traveling though the spinal nerves are not involved in triggering the swallowing reflex. They are believed to be involved in providing sensation from the esophageal region^9^.

According to the previous studies, the SLN plays an important role in triggering the swallowing reflex^1,2^. Mechanical stimulation of the SLN-innervated regions and electrical stimulation of the SLN can readily trigger the swallowing reflex^2,10^. Furthermore, local anesthesia of the SLN in healthy individuals increases the incidence of penetration/aspiration of boli into the airway, the amount of pharyngeal residues of the boli, effortful swallowing, and the illusory globus sensation in the throat during swallowing^11,12^.

Transient receptor potential ankyrin 1 channels (TRPA1s) are Ca^2+^-permeable non-selective cation channels widely expressed in sensory neurons and in non-neuronal cells^13^. TRPA1s can act as chemosensors and are activated by a wide range of chemical compounds including natural agents such as allyl isothiocyanate (AITC; present in mustard oil, wasabi, and horseradish), cinnamaldehyde (present in cinnamon), and allicin (present in garlic^14–16^. Previous animal study reported TRPA1 expression in the NPJc^17^, while a human biopsy study reported TRPA1 expression in the pharyngeal and laryngeal regions^18^. The presence of TRPA1s in the swallowing-related regions and ganglia suggests potential roles in the swallowing reflex. TRPA1s were also reported to act as cold/noxious cold sensors (activated at temperatures < 17 °C)^15,19^ and as mechanosensors^20–22^, although these functions remain controversial^13,23^. To the best of our knowledge, no studies have systematically investigated whether TRPA1s act as chemosensors, cold sensors, or mechanosensors in triggering of the swallowing reflex. Understanding the role of TRPA1s in triggering of the swallowing reflex is important for developing therapeutics to manage dysphagia. In this context, a recent clinical study reported that TRPA1 agonists improved the swallowing response in patients with oropharyngeal dysphagia associated with aging, stroke, and neurodegenerative diseases^24^.

At present, there are contrasting findings on the effects of cold thermal stimuli applied to the swallowing-related regions, with reports of improvement of swallowing responses and of no effects^25–32^. Importantly, however, those studies did not measure the on-site temperature changes in the swallowing-related regions after application of the cold thermal stimuli. The initial temperature reductions following application of cold stimuli may rapidly resolve because of the high mucosa vascularity in those regions. Thus, recording the on-site temperature changes after application of cold stimuli is essential when examining the effects of cold temperature on swallowing. In the present study, we examined the on-site temperature changes and effects on the swallowing reflex following application of cold stimuli in the laryngopharyngeal and associated laryngeal regions.

The aim of this study was to examine whether TRPA1s act as chemosensors, cold sensors, or mechanosensors to trigger the swallowing reflex and determine the effects of cold stimuli on triggering of this reflex.

## Results

### TRPA1s are predominantly localized on thin nerve fibers and fibroblast-like cells in the SLN-innervated swallowing-related regions

**Figure 1:**
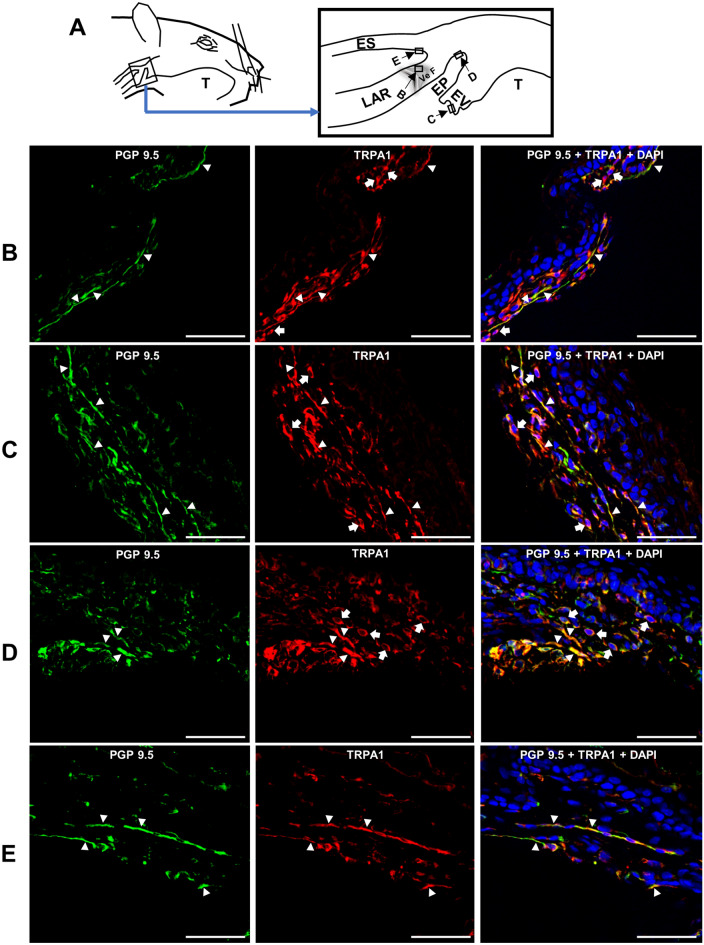
TRPA1s are localized on thin nerve fibers and fibroblast-like cells in the laryngopharyngeal and associated laryngeal regions. (**A**) Schematics of the laryngopharyngeal and associated laryngeal regions. Rectangles with arrows and letters show the regions where the photomicrographs were taken. Photomicrographs of TRPA1 localization in the (**B**) vestibular fold (Ve F), (**C**) epiglottic vallecula (EV), (**D**) epiglottis (EP), and (**E**) cervical esophagus (ES). White arrowheads indicate examples of TRPA1 expression on PGP 9.5-expressing nerve fibers. White arrows indicate examples of TRPA1 expression on fibroblast-like cells. Scale bars = 50 μm. LAR, larynx; T, tongue.

Photomicrographs of TRPA1 expression in different SLN-innervated swallowing-related regions are shown in Fig. 1. Protein gene product (PGP) 9.5 was used to detect nerve fibers and 4ʹ,6-diamidino-2-phenylindole (DAPI) was used for cell nucleus staining. TRPA1s were predominantly expressed on some thin nerve fibers and connective tissue cells (likely fibroblasts) present in the subepithelial connective tissue regions (Fig. 1). TRPA1s were also expressed on some chondrocyte-like cells in the epiglottic cartilage (not shown). No TRPA1 immunoreactivity (IR) was observed on sensory corpuscle-like nerve structures or on thick nerve fibers present in these regions (Supplemental Fig. 2).

**Figure 2:**
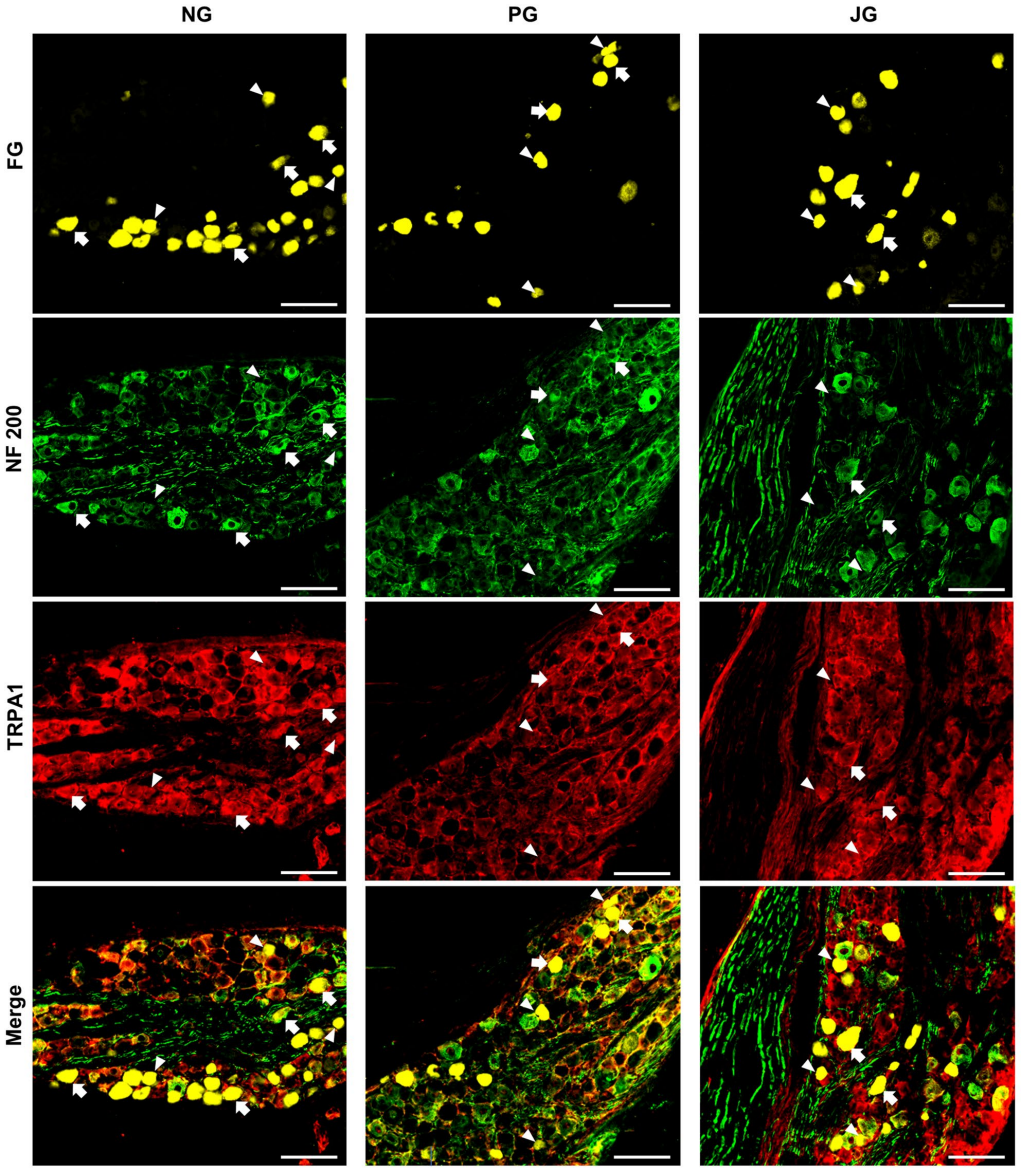
Photomicrographs of TRPA1 localization in the NPJc. TRPA1 expression in the NG, PG, and JG. White arrows indicate examples of cells positive for FG, TRPA1, and NF-200. White arrowheads indicate examples of cells positive for both FG and TRPA1, but negative for NF-200. Scale bars = 100 μm.

### TRPA1s are predominantly expressed on small- to medium-sized SLN-afferent neurons in the NPJc

**Figure 3:**
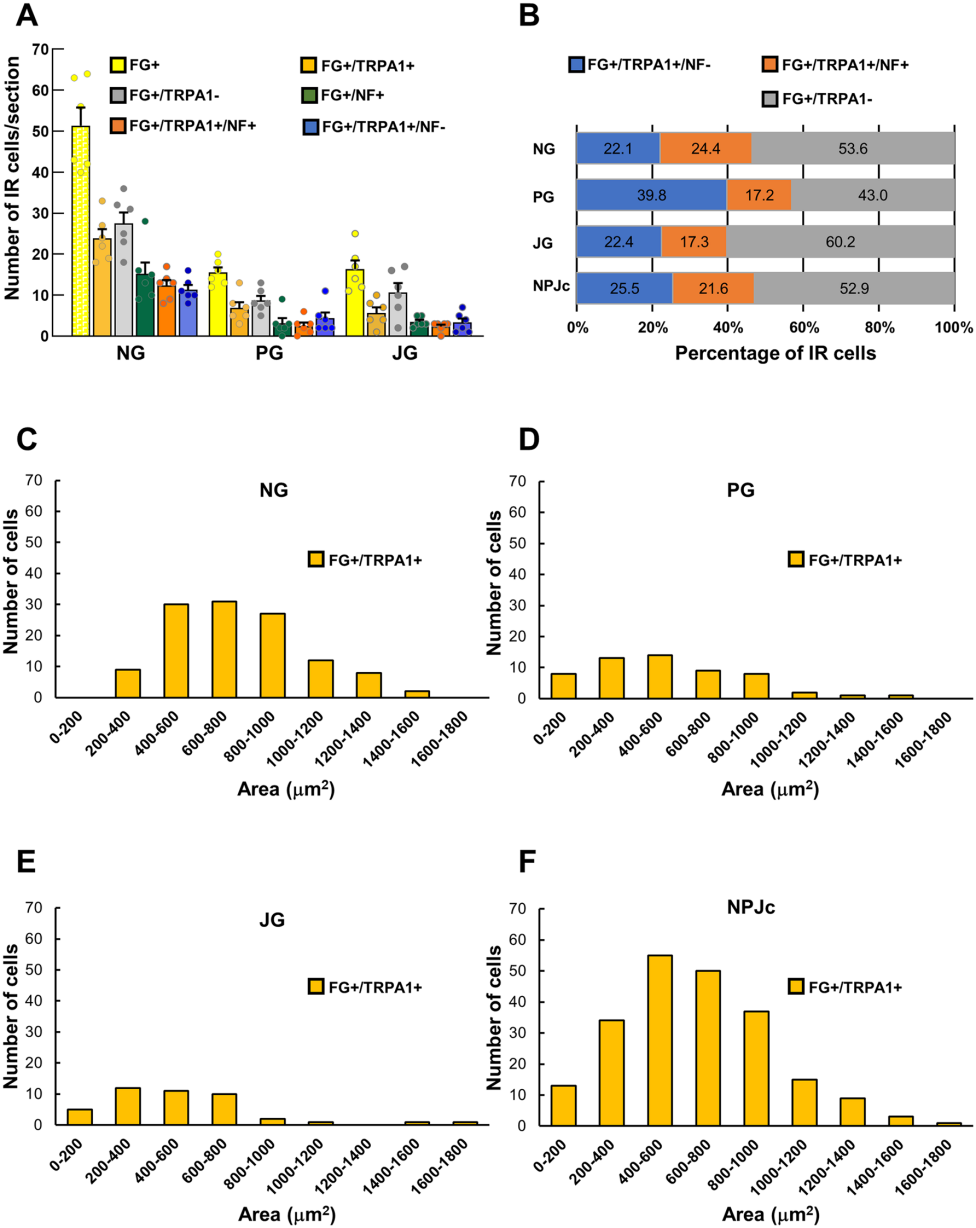
Distribution of TRPA1 localization in the NPJc. (**A**) Number of TRPA1-positive cells/section in the NG, PG, and JG. (**B**) Percentage of FG-stained, TRPA1-positive cells on myelinated (FG + /TRPA1 + /NF +) and unmyelinated (FG + /TRPA1 + /NF −) neurons. (**C**) Size (area) distribution of TRPA1-positive cells in the NG. (**D**) Size (area) distribution of TRPA1-positive cells in the PG. (**E**) Size (area) distribution of TRPA1-positive cells in the JG. (**F**) Size (area) distribution of TRPA1-positive cells in the whole NPJc. FG + , cells stained with FG; FG + /TRPA1 + , FG-stained cells immunopositive for TRPA1; FG + /TRPA1 − , FG-stained cells immunonegative for TRPA1; FG + /NF + , FG-stained cells immunopositive for NF-200; FG + /TRPA1 + /NF + , FG-stained cells immunopositive for TRPA1 and NF-200; FG + /TRPA1 + /NF − , FG-stained cells immunopositive for TRPA1 but not NF-200. *n* = 6. Data in (**A**) are presented as mean ± SEM. Circles in column graph (**A**) represent individual data points. IR cells were counted using ImageJ software. Cell counts were performed in the sections showing the highest number of TRPA1-IR cells. Three sections were used from each rat (one section/ganglion).

Next, we traced SLN-afferent neurons in the NPJc using the retrograde tracer fluoro-gold (FG) (Fig. 2). TRPA1s were expressed on cell bodies located in the nodose (NG), petrosal (PG), and jugular (JG) ganglia (Fig. 2; Fig. 3A), including on approximately 50% of FG-stained SLN-afferent neurons (Fig. 3B). The majority of TRPA1s were expressed on small- to medium-sized SLN-afferent neurons (small neurons, 49.1%; medium neurons, 45.3%; Fig. 3C, D, E, and F; Table 1), with only limited expression on large SLN-afferent neurons (5.6%; Table 1).

**Table 1:** Cell size distribution of FG stained TRPA1-IR neurons in the NPJc.

	Small (0–600 μm^2^)	Medium (600–1200 μm^2^)	Large (> 1200 μm^2^)
NG	35.3% (42/119)	58.0% (69/119)	6.7% (8/119)
PG	67.3% (35/52)	38.9% (15/52)	3.8% (2/52)
JG	65.1% (28/43)	30.2% (13/43)	4.7% (2/43)
NPJc	49.1% (105/214)	45.3% (97/214)	5.6% (12/214)

Average area of FG stained TRPA1-IR cell bodies (mean ± SEM)			
	Small (0–600 μm^2^)	Medium (600–1200 μm^2^)	Large (> 1200 μm^2^)
NG	417.3 ± 104.15 (42)	826.1 ± 169.92 (69)	1351.1 ± 39.17 (8)
PG	344.7 ± 143.53 (35)	808.1 ± 142.81 (15)	1385.0 ± 171.38 (2)
JG	353.0 ± 140.86 (28)	764.2 ± 173.96 (13)	1643.4 ± 46.90 (2)
NPJc	376.0 ± 132.34 (105)	815.0 ± 166.29 (97)	1405.4 ± 47.59 (12)

All data obtained from 18 Sects. (1 section/ganglion/rat). *n* = 6. The number within each parenthesis indicates the raw number of analyzed neurons.

Neurofilament (NF)-200 was used to differentiate between myelinated and unmyelinated neurons. In the whole NPJc, TRPA1s were expressed slightly more on unmyelinated neurons (NF-200 negative) than on myelinated neurons (NF-200 positive) (Fig. 3B).

### A chemical agonist of TRPA1s dose-dependently triggered swallowing reflexes

**Figure 4:**
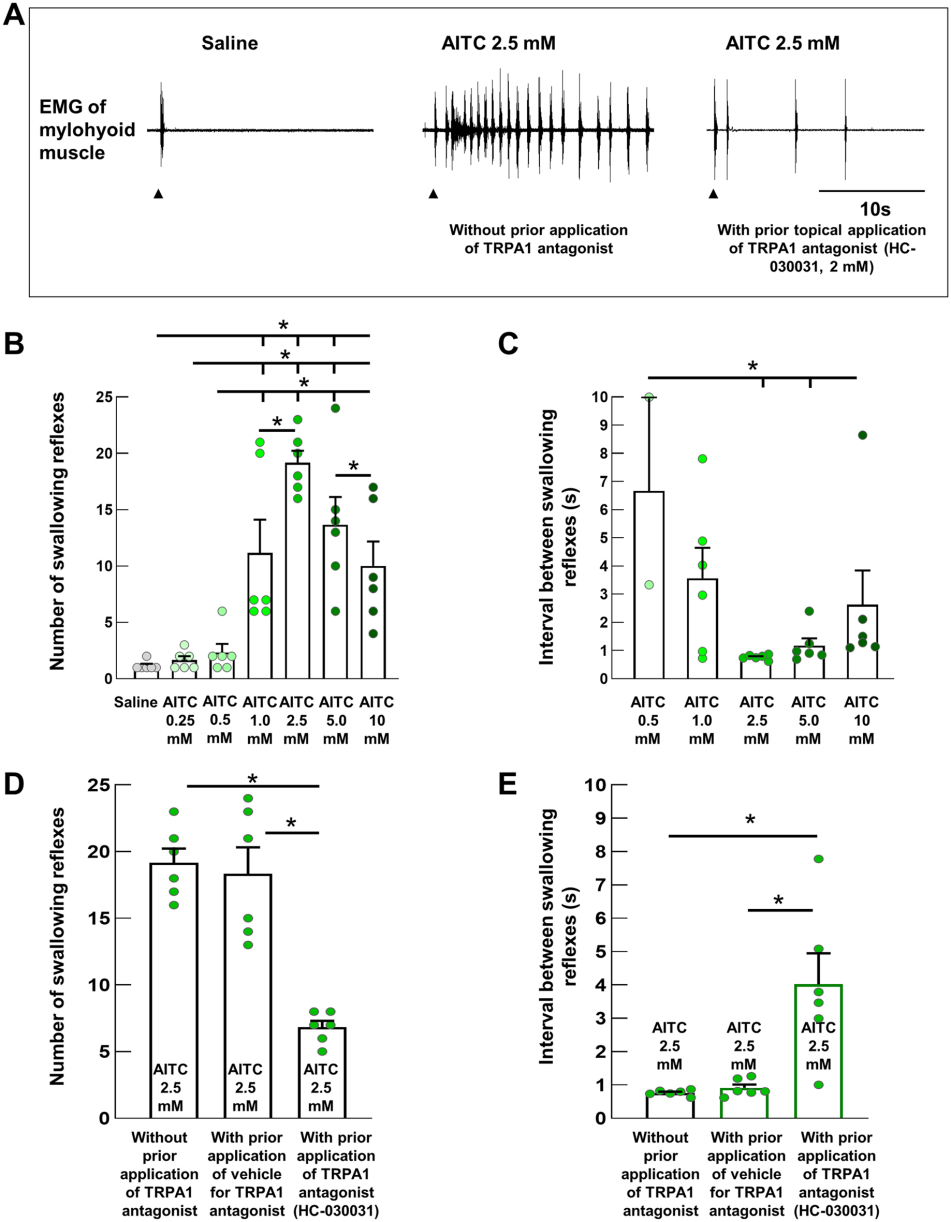
Topical application of AITC, a chemical agonist of TRPA1s triggered the swallowing reflexes which were significantly attenuated by prior topical application of a TRPA1 antagonist. (**A**) Swallowing reflexes indicated by high amplitude EMG activity in the mylohyoid muscle triggered by saline and AITC (2.5 mM) (with and without prior topical application of a TRPA1 antagonist). Black arrowheads indicate the onset of stimulating solution delivery. (**B**) Comparison of the number of swallowing reflexes triggered by saline and different AITC concentrations. (**C**) Comparison of the intervals between the swallowing reflexes triggered by different AITC concentrations. (**D**) Comparison of the numbers of swallowing reflexes triggered by AITC with and without prior application of the TRPA1 antagonist or vehicle. (**E**) Comparison of the intervals between the swallowing reflexes triggered by AITC with and without prior application of the TRPA1 antagonist or vehicle. *n* = 6. The number of triggered swallowing reflexes counted for 20 s following application of the stimulating solutions and the intervals between the swallowing reflexes calculated from the reflexes evoked within the 10-s time period following the onset of stimulating solution delivery. Data are presented as mean ± SEM. Circles in the column graphs represent individual data points. In (**C**), there are only two individual data points for AITC 0.5 mM because only two rats showed triggering of more than one swallowing reflex (within 10-s time period) at this concentration (at least two swallowing reflexes are required to measure the interval between the reflexes). *P < .05 by one-way repeated measures ANOVA followed by Tukey’s test or Kruskal–Wallis one-way ANOVA on ranks followed by Tukey’s test. S, seconds.

To examine whether activation of TRPA1s can trigger the swallowing reflex, we stimulated the swallowing-related regions with different concentrations of room temperature AITC (diluted in saline) delivered as a single bolus dose (50 *μ*L) over 1 s (Fig. 4). The regions were also stimulated with saline (vehicle for AITC), with only one or two swallowing reflexes triggered immediately following saline delivery. Delivery of AITC triggered swallowing reflexes in a dose-dependent manner up to 2.5 mM AITC. The highest number of triggered reflexes (19.17 ± 1.08) was observed at 2.5 mM AITC (Fig. 4B). Increasing the AITC concentration to 10 mM significantly reduced the number of triggered reflexes (10.00 ± 2.16) compared with that at 2.5 mM. The number of swallowing reflexes at 1 mM, 2.5 mM, 5 mM, and 10 mM AITC was significantly higher than that triggered by 0.25 mM and 0.5 mM AITC or saline (Fig. 4B). The intervals between the triggered reflexes were shortened by increasing the concentration of AITC and the shortening of the intervals was most prominent within the early time period following the onset of AITC delivery. We calculated the average interval between the swallowing reflexes from the reflexes evoked within the 10-s time period following the onset of stimulating solution delivery. The intervals between the triggered reflexes at 2.5 mM (0.77 ± 0.03 s), 5 mM (1.18 ± 0.25 s), and 10 mM (2.63 ± 1.21 s) AITC were significantly shorter than that at 0.5 mM AITC (6.66 ± 1.92 s) (Fig. 4C).

### Prior topical application of the TRPA1 antagonist significantly reduced the triggering of AITC-induced swallowing reflexes

Prior topical application of the TRPA1 antagonist HC-030031 (2 mM), but not the vehicle solution for the antagonist (dimethyl sulfoxide [DMSO] and Tween-80 dissolved in saline), significantly reduced the number of AITC (2.5 mM)-induced swallowing reflexes (19.17 ± 1.08 and 6.83 ± 0.48 with and without prior application of the TRPA1 antagonist, respectively) (Fig. 4D). Additionally, the TRPA1 antagonist significantly increased the intervals between the triggered reflexes (0.77 ± 0.03 s and 4.02 ± 0.93 s with and without prior application of the TRPA1 antagonist, respectively) (Fig. 4E).

### Topical single bolus application of cold solutions briefly reduced the on-site temperature to levels at which TRPA1s can be activated, but had no effect on triggering of swallowing reflexes

**Figure 5:**
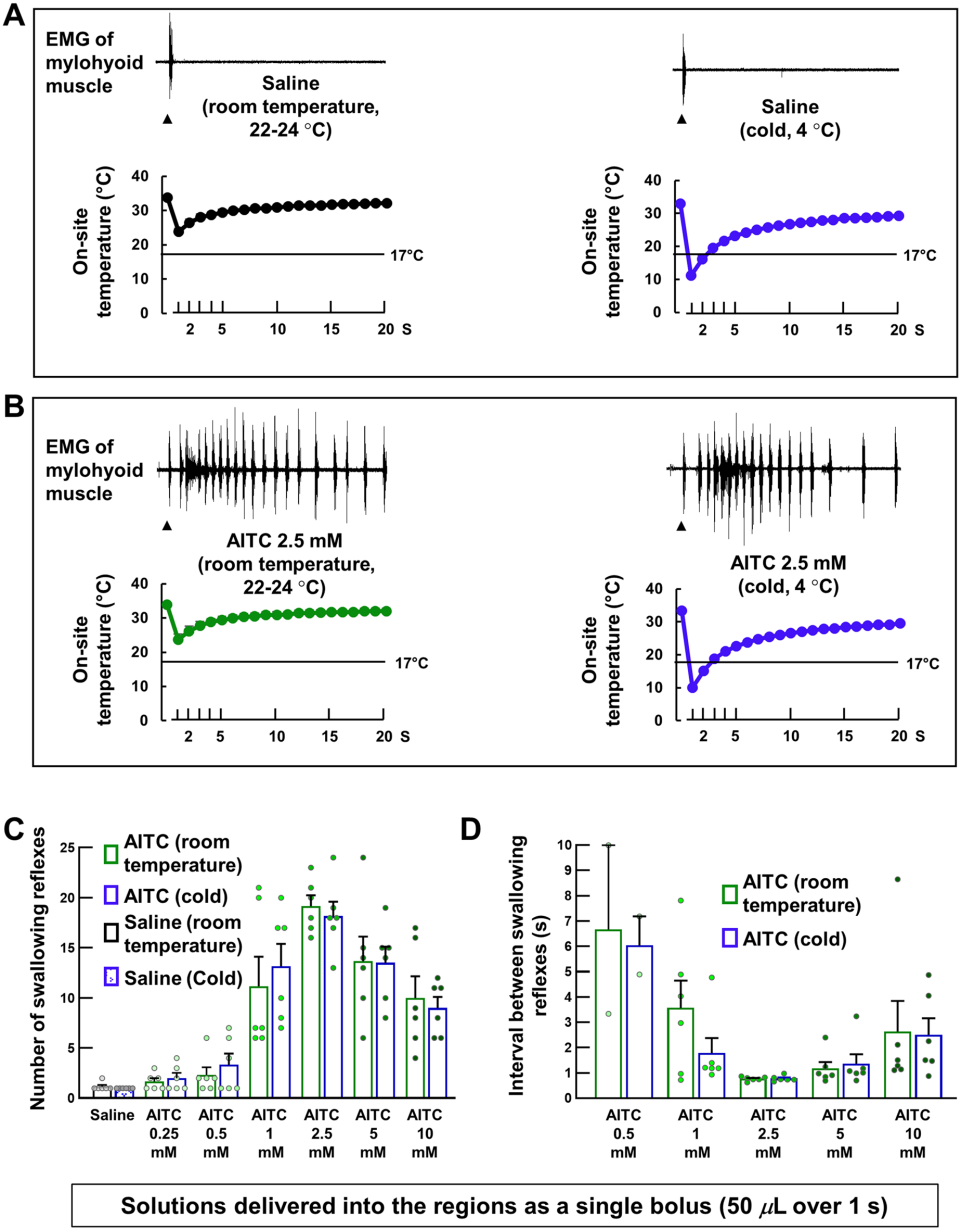
Topical application of cold solutions as a single bolus very briefly reduced the on-site temperature to levels at which TRPA1s can be activated, but had no effect on triggering of the swallowing reflex because of the rapid increase in the on-site temperature. (**A**) The triggered swallowing reflexes and changes in the on-site temperature following delivery (50 *μ*L, single bolus) of saline at cold (4 °C) and room (22–24 °C) temperatures. Black arrowheads indicate the onset of stimulating solution delivery. Data (mean ± SEM) for the on-site temperature are from a separate cohort of seven rats not used for counting the swallowing reflexes. (**B**) The triggered swallowing reflexes and changes in the on-site temperature following delivery (50 *μ*L, single bolus) of AITC at cold (4 °C) and room (22–24 °C) temperatures. Black arrowheads indicate the onset of stimulating solution delivery. Data (mean ± SEM) for the on-site temperature are from a separate cohort of seven rats not used for counting the swallowing reflexes. (**C**) The number of swallowing reflexes triggered by delivery (50 *μ*L, single bolus) of saline and different AITC concentrations at cold (4 °C) and room (22–24 °C) temperatures. (**D**) The intervals between the swallowing reflexes triggered by delivery (50 *μ*L, single bolus) of saline and different AITC concentrations at cold (4 °C) and room (22–24 °C) temperatures. *n* = 6. The number of triggered swallowing reflexes counted for 20 s following application of the stimulating solutions and the intervals between the swallowing reflexes calculated from the reflexes evoked within the 10-s time period following the onset of stimulating solution delivery. Data are presented as mean ± SEM. Circles in the column graphs represent individual data points. In (**D**), there are only two individual data points for AITC 0.5 mM because only two rats showed triggering of more than one swallowing reflex (within 10-s time period) at this concentration (at least two swallowing reflexes are required to measure the interval between the reflexes). There were no differences between the solutions at cold and room temperatures (paired *t*-test or Wilcoxon’s signed rank test).

We conducted a series of experiments to examine the effect of putative TRPA1 activation by cold stimuli on triggering of the swallowing reflex. First, we stimulated the swallowing-related regions with saline at cold (4 °C) and room (22–24 °C) temperatures (Fig. 5A). To determine the on-site temperature changes after saline delivery, we recorded the on-site temperature using a fine surface temperature sensor placed on the mucosa of the regions in a separate cohort of rats (*n* = 7, see “Methods” section). The lower traces in Fig. 5A show the changes in on-site temperature following delivery of saline at cold and room temperatures. Following cold saline delivery, the on-site temperature decreased immediately (< 17 °C for 2.19 ± 0.13 s), but then rapidly increased toward pre-delivery levels (Fig. 5A). Despite this brief temperature reduction to levels at which TRPA1s can be activated, there was no change in the number of triggered swallowing reflexes compared with room temperature saline (Fig. 5A, C). Note that the on-site temperature did not fall to levels at which TRPA1s can be activated (< 17 °C) following delivery of room temperature saline.

Next, we applied different concentrations of cold AITC (4 °C) to examine the effect of putative activation of TRPA1s using a combination of cold thermal and chemical stimuli on triggering of the swallowing reflex. The on-site temperature changes following delivery of cold and room temperature AITC (Fig. 5B) were similar to those for delivery of cold and room temperature saline, respectively. Despite the brief reduction in the on-site temperature to < 17 °C following cold AITC delivery (Fig. 5B), there were no differences in the number of reflexes triggered by different concentrations of cold AITC compared with those using room temperature AITC (Fig. 5C). Additionally, there were no differences in the reflex intervals between the cold and room temperature AITC at each concentration (Fig. 5D).

### Increasing the time that the on-site temperature remained at levels at which TRPA1s can be activated reduced the frequency of triggering of AITC-induced swallowing reflexes

**Figure 6:**
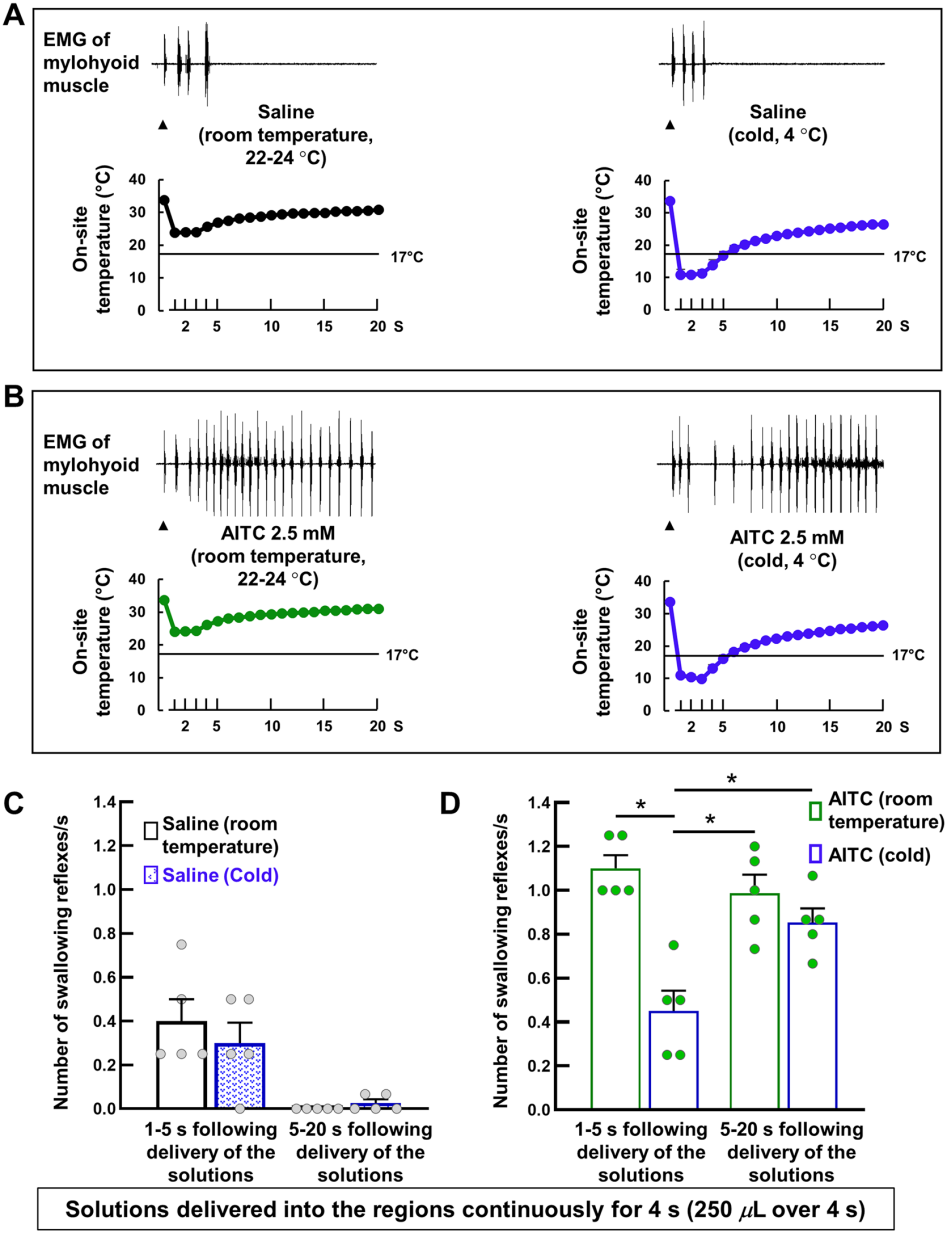
Continuous topical application of cold solutions increased the time that the on-site temperature was reduced to levels at which TRPA1s can be activated, which paradoxically reduced the triggering of AITC-induced swallowing reflexes. (**A**) The triggered swallowing reflexes and changes in the on-site temperature with continuous delivery of saline (250 *μ*L over 4 s) at cold (4 °C) and room (22–24 °C) temperatures. Black arrowheads indicate the onset of stimulating solution delivery. Data (mean ± SEM) for the on-site temperature are from a separate cohort of seven rats not used for counting the swallowing reflexes. (**B**) The triggered swallowing reflexes and changes in the on-site temperature with continuous delivery of AITC (250 *μ*L over 4 s) at cold (4 °C) and room (22–24 °C) temperatures. Black arrowheads indicate the onset of stimulating solution delivery. Data (mean ± SEM) for the on-site temperature are from a separate cohort of seven rats not used for counting the swallowing reflexes. (**C**) The frequency of swallowing reflexes/second during the time period of 1–5 s triggered by continuous saline delivery (250 *μ*L over 4 s) at cold (4 °C) and room (22–24 °C) temperatures. There were no differences between the solutions at cold and room temperatures (Wilcoxon’s signed rank test). (**D**) The frequency of swallowing reflexes/second during the time periods of 1–5 s and 5–20 s triggered by continuous AITC delivery (250 *μ*L over 4 s) at cold (4 °C) and room (22–24 °C) temperatures. *n* = 5. Data are presented as mean ± SEM. Circles in the column graphs represent individual data points. *P < .05 by one-way repeated measures ANOVA followed by Tukey’s test.

In the two previous experiments, the on-site temperature was reduced < 17 °C very briefly (Fig. 5A and B) following single bolus delivery of cold saline/AITC (50 *μ*L over 1 s). To increase the time that the on-site temperature was < 17 °C, we delivered 250 *μ*L of cold (4 °C) saline/AITC (2.5 mM) into the swallowing-related regions over 4 s (Fig. 6). We also examined triggering of the swallowing reflex by delivery of 250 *μ*L of room temperature saline/AITC (2.5 mM) (Fig. 6).

Delivery of cold solutions continuously for 4 s maintained the on-site temperature < 17 °C for a longer time (4.88 ± 0.27 s) than that for the 1-s delivery (single bolus delivery) (Fig. 6A and B). By contrast, delivery of room temperature solutions did not reduce the on-site temperature < 17 °C (Fig. 6A and B). We then calculated the frequency of the triggered reflexes/second over the time periods of 1–5 s and 5–20 s following the onset of solution delivery. The time periods were chosen based on the reduction of on-site temperature following cold (4 °C) solution delivery. The on-site temperature was ≤ 17 °C during the 1–5-s time period, but was ≥ 17 °C during the 5–20-s time period. After initiating delivery of 250 μL of cold or room temperature saline over 4 s, there was an increase in the number of triggered reflexes (Fig. 6A) compared with that for single bolus delivery of 50 μL of the cold or room temperature solutions (Fig. 5A). In the majority of animals, swallowing reflexes were triggered within 5 s after delivery of cold or room temperature saline (Fig. 6A and C). Two animals showed an additional swallowing reflex triggered after 5 s following cold saline delivery.

There were no differences in the total number of triggered reflexes (over 20 s) between delivery of cold or room temperature saline (3.40 ± 0.51 vs 3.00 ± 0.45, respectively). Additionally, there were no differences in the frequency of triggered reflexes/second between the delivery of cold and room temperature saline (Fig. 6C). By contrast, the total number of triggered reflexes (over 20 s) was significantly reduced following cold AITC (2.5 mM) delivery compared with that for room temperature AITC (2.5 mM) (16.60 ± 0.93 vs 20.80 ± 1.24, respectively). The frequency of triggered reflexes/second during the 1–5-s time period was significantly lower for cold AITC (when the on-site temperature was ≤ 17 °C) compared with that for room temperature AITC (when the on-site temperature was > 17 °C) (Fig. 6D). Additionally, upon cold AITC delivery the frequency of the triggered swallowing reflexes/second was significantly lowered during the 1–5-s time period (when the on-site temperature was ≤ 17 °C) compared with that for the 5–20-s time period (when the on-site temperature was ≥ 17 °C) (Fig. 6D).

### Prolong reduction of on-site temperature to levels at which TRPA1s can be activated prevented the triggering of AITC-induced swallowing reflexes

**Figure 7:**
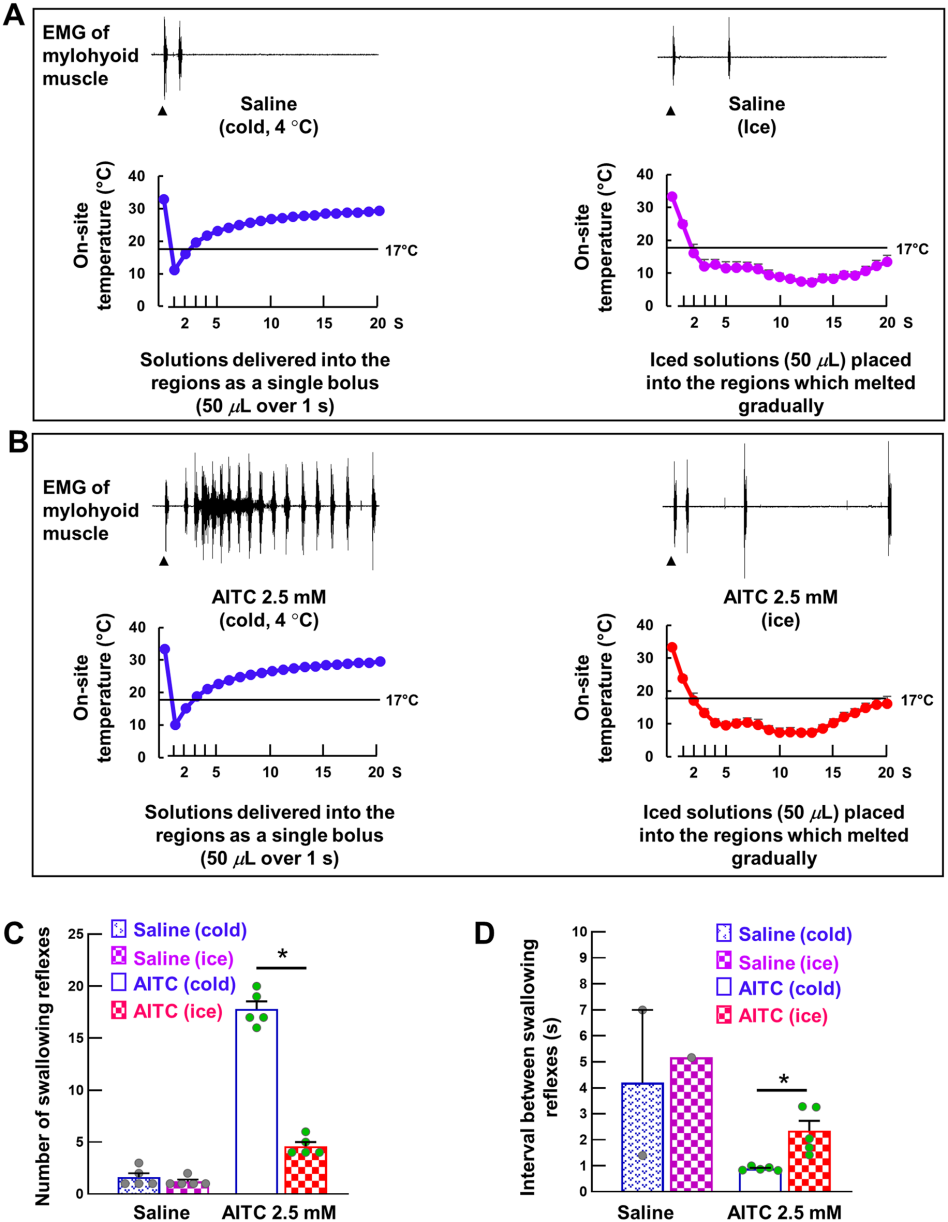
Topical application of iced solutions persistently reduced the on-site temperature to levels at which TRPA1s can be activated, which prevented the triggering of swallowing reflexes by AITC. (A) Triggered swallowing reflexes and changes in the on-site temperature by delivery of cold (4 °C) saline (50 *μ*L, single bolus) and placement of iced saline (50 *μ*L solution). Black arrowheads indicate the onset of stimulating solution delivery/onset of iced solution placement. Data (mean ± SEM) for the on-site temperature are from a separate cohort of seven rats not used for counting the swallowing reflexes. (**B**) Triggered swallowing reflexes and changes in the on-site temperature by delivery of cold (4 °C) AITC (50 *μ*L, single bolus) and placement of iced AITC (50 *μ*L solution). Black arrowheads indicate the onset of stimulating solution delivery/onset of iced solution placement. Data (mean ± SEM) for the on-site temperature are from a separate cohort of seven rats not used for counting the swallowing reflexes. (**C**) The number of swallowing reflexes triggered by delivery of cold (50 *μ*L, single bolus) and iced (50 *μ*L solution) saline/AITC. (**D**) The intervals of the triggered swallowing reflexes by delivery of cold (50 *μ*L, single bolus) and iced (50 *μ*L solution) saline/AITC. *n* = 5. The number of triggered swallowing reflexes counted for 20 s following application of the stimulating solutions and the intervals between the swallowing reflexes calculated from the reflexes evoked within the 10-s time period following the onset of stimulating solution delivery. Data are presented as mean ± SEM. Circles in the column graphs represent individual data points. In (**D**), there are only one/two individual data points for saline because only one/two rats showed triggering of more than one swallowing reflex (within 10-s time period) for saline (at least two swallowing reflexes are required to measure the interval between the reflexes). *P < .05 by paired *t*-test or Wilcoxon’s signed rank test.

In these experiments, we placed iced saline/AITC (2.5 mM; 50 *μ*L) into the swallowing-related regions. Gradual melting of the iced solutions kept the on-site temperature < 17 °C for prolonged times (Fig. 7A and B), which allowed us to examine the effect of longer cold stimulation on triggering of the swallowing reflex (Fig. 7). We compared the effect of placement of the iced solutions (50 μL) on triggering of the swallowing reflex with that for delivery of cold (4 °C) solutions (50 μL). Despite a marked difference in the on-site temperatures, there were no differences in the number of triggered swallowing reflexes between iced and cold (4 °C) saline (1.20 ± 0.20 vs 1.60 ± 0.40, respectively; Fig. 7C). By contrast, the number of swallowing reflexes was markedly reduced (4.4 ± 0.51 vs 17.80 ± 0.73, respectively) and the intervals between the triggered reflexes significantly increased (2.34 ± 0.39 s vs 0.89 ± 0.03 s, respectively) following placement of iced AITC compared with delivery of cold AITC (Fig. 7C and D).

### Prior topical application of the TRPA1 antagonist at a concentration that significantly reduced the triggering of AITC-induced swallowing reflexes had no effect on the threshold for mechanical stimuli-induced swallowing reflexes

**Figure 8:**
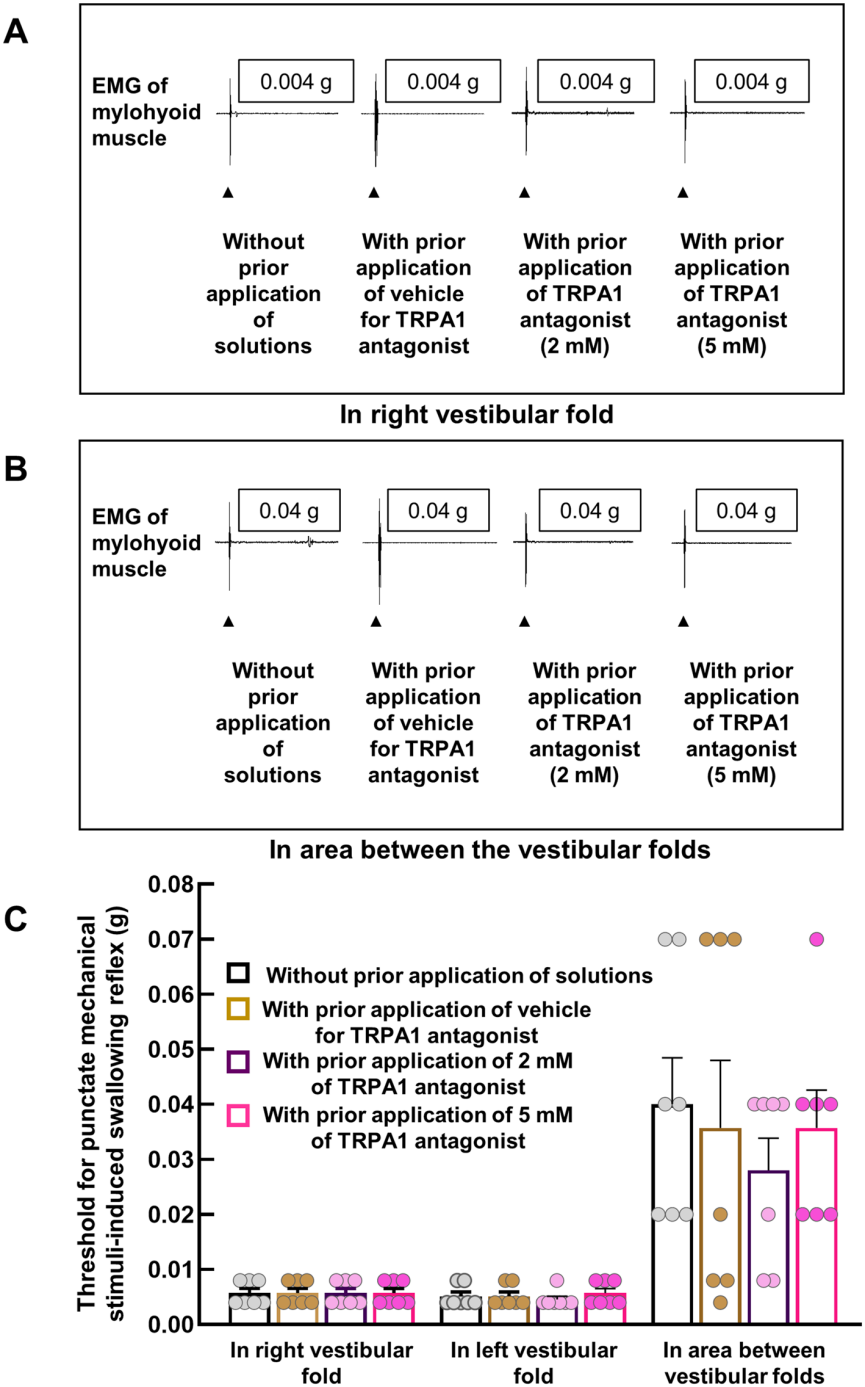
Prior topical application of the TRPA1 antagonist at a concentration that effectively reduced AITC-induced reflexes had no effect on the threshold for triggering the swallowing reflex by punctate mechanical stimuli. (**A**) Representative figures of swallowing reflexes triggered by threshold-level force (shown in g) applied on the right vestibular fold by von-Frey filaments with or without prior application of the TRPA1 antagonist/vehicle. Black arrowheads indicate the onset of the punctate mechanical stimuli by the von-Frey filaments. (**B**) Representative figures of swallowing reflexes triggered by threshold-level force (shown in g) applied on the area between the vestibular folds by von-Frey filaments with or without prior application of the TRPA1 antagonist/vehicle. Black arrowheads indicate the onset of the punctate mechanical stimuli by the von-Frey filaments. (**C**) Threshold to trigger a swallowing reflex by punctate mechanical stimuli on the vestibular folds (right and left) and on the area between the vestibular folds with or without prior application of the TRPA1 antagonist/vehicle. The TRPA1 antagonist was used at either 2 mM (a concentration that significantly reduced the triggering of the swallowing reflex by AITC) or 5 mM (more than double the concentration that significantly reduced the triggering of the swallowing reflex by AITC). *n* = 7. Data are presented as mean ± SEM. Circles in column graphs represent individual data points. There were no differences between with and without prior application of the TRPA1 antagonist/vehicle (Friedman repeated measures ANOVA on ranks).

To examine the contribution of TRPA1s in the mechanical stimuli-induced swallowing reflex, we performed several experiments using the TRPA1 antagonist HC-030031 (which significantly reduced triggering of the AITC-induced reflexes). First, we examined the effect of prior topical application of the TRPA1 antagonist on the threshold to trigger a swallowing reflex by punctate mechanical stimuli. This threshold was measured using von-Frey filaments in three sites within the SLN-innervated swallowing-related regions—the right vestibular fold, left vestibular fold, and the midline area between these folds. The threshold to trigger a swallowing reflex by punctate mechanical stimuli in the vestibular folds (both right and left) was very low (Fig. 8). To examine the effect of blocking TRPA1s on the threshold to evoke a swallowing reflex, the TRPA1 antagonist was applied topically to the swallowing-related region at 10 min before the mechanical stimuli. An antagonist concentration (2 mM) that significantly reduced the number of AITC induced-swallowing reflexes (Fig. 4) had no effect on the threshold to trigger a swallowing reflex at any of the measuring sites compared with the no antagonist and antagonist vehicle groups (Fig. 8). Similarly, increasing the concentration of the antagonist to 5 mM (more than double the concentration that reduced AITC induced-swallowing reflexes) had no effect on the threshold to evoke a swallowing reflex by punctate mechanical stimuli (Fig. 8).

### Prior topical application of the TRPA1 antagonist had no effect on the triggering of low-force or high-force mechanical pressure stimuli-induced swallowing reflexes

**Figure 9:**
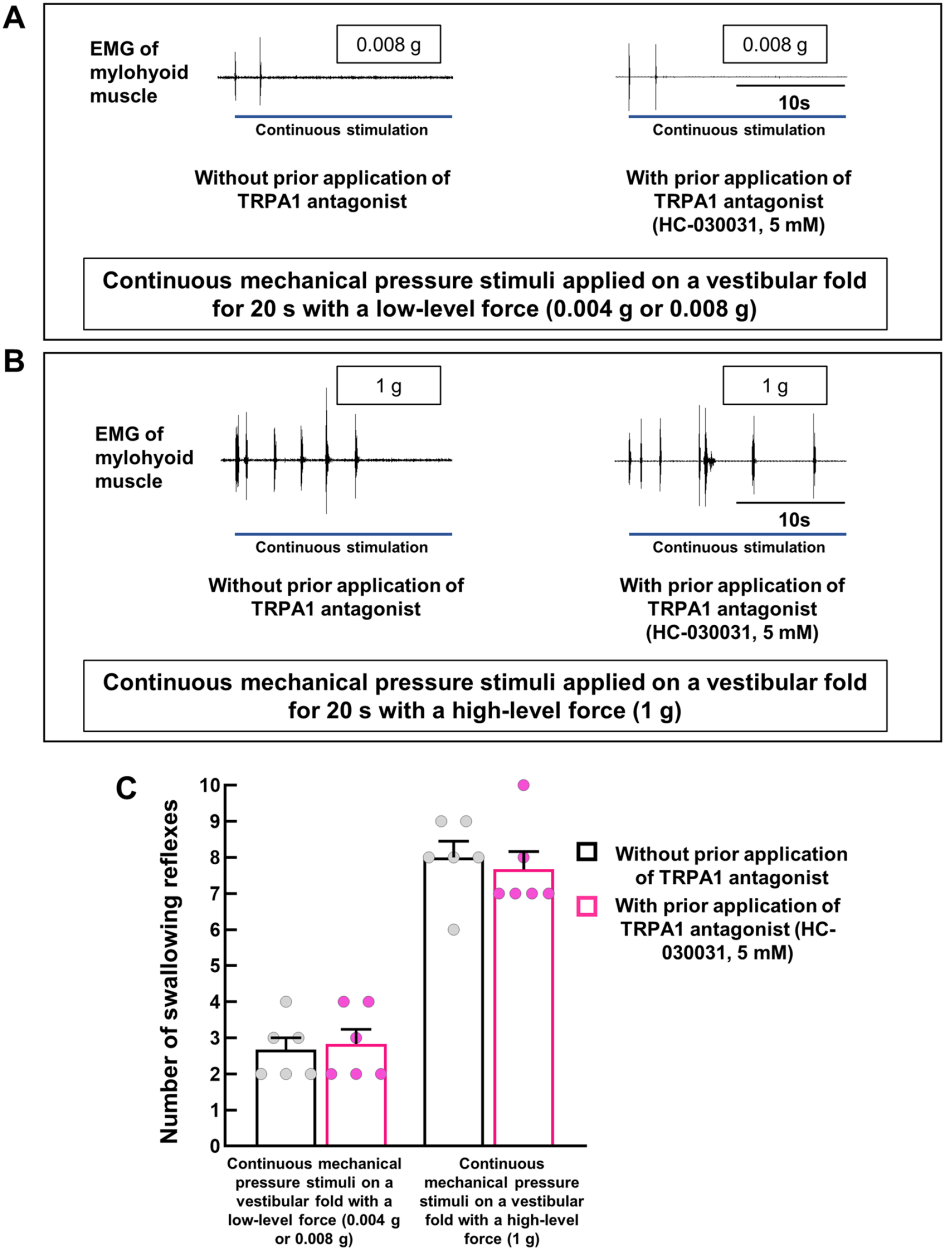
Prior topical application of the TRPA1 antagonist had no effect on triggering of the continuous low-force or high-force mechanical pressure stimuli-induced swallowing reflex. (**A**) Representative figures of swallowing reflexes triggered by continuous mechanical-pressure stimuli with a low-level force (threshold-level force) applied on a vestibular fold by von-Frey filaments (0.008 g) with or without prior application of the TRPA1 antagonist. Blue solid lines indicate the duration of continuous mechanical pressure stimuli applied by the von-Frey filaments. (B) Representative figures of swallowing reflexes triggered by continuous mechanical-pressure stimuli with a high-level force (approximately 125–250 times higher than the threshold-level force) applied on a vestibular fold by von-Frey filaments (1 g) with or without prior application of the TRPA1 antagonist. Blue solid lines indicate the duration of continuous mechanical pressure stimuli applied by the von-Frey filaments. (**C**) Number of triggered swallowing reflexes by continuous mechanical pressure stimuli with low-level and high-level forces on a vestibular fold (right/left) with or without prior application of the TRPA1 antagonist. The concentration of the TRPA1 antagonist was more than twice that which significantly reduced the triggering of the swallowing reflex by AITC. *n* = 6. Data are presented as mean ± SEM. Circles in the column graphs represent individual data points. There were no differences between with and without prior application of the TRPA1 antagonist (paired *t*-test/Wilcoxon’s signed rank test).

Next, we applied continuous mechanical pressure stimuli using von-Frey filaments in the vestibular folds (right/left) for 20 s to evoke a mechanical pressure stimuli-induced swallowing reflex. To examine the role of TRPA1s in triggering of the swallowing reflex by continuous low-force mechanical pressure stimuli, a continuous threshold-level force was applied to the right/left vestibular fold for 20 s and the number of triggered reflexes was compared between with and without prior topical application of the TRPA1 antagonist (5 mM; more than double the concentration that reduced the AITC induced-swallowing reflexes) (Fig. 9). Application of continuous low-force mechanical pressure stimuli triggered a few swallowing reflexes (Fig. 9A). There were no differences in the number of low-force mechanical pressure stimuli-induced reflexes between with and without prior application of the TRPA1 antagonist (Fig. 9A and C).

To activate high-threshold mechanoreceptors and to assess the contribution of TRPA1s in triggering the swallowing reflex by continuous high-force mechanical pressure stimuli, a 1-g von-Frey filament force (approximately 125–250 times higher than the threshold-level force required to trigger a swallowing reflex) was applied to the right/left vestibular folds for 20 s and the number of triggered swallowing reflexes with and without prior application of the TRPA1 antagonist (5 mM) were counted. The continuous high-force mechanical pressure stimuli in the vestibular folds triggered numerous swallowing reflexes (Fig. 9B). However, there were no differences in the number of reflexes between with and without prior application of the TRPA1 antagonist (Fig. 9B and C).

### Prior topical application of the TRPA1 antagonist had no effect on the triggering of swallowing reflexes induced by delivery of saline continuously for 4 s

We also examined the effect of the TRPA1 antagonist on the number and interval of the swallowing reflexes induced by delivery of saline (250 *μ*L at room temperature) continuously for 4 s to assess the contribution of TRPA1s in saline-induced swallowing reflexes. There were no differences in the number and interval of triggered reflexes between with and without prior application of the TRPA1 antagonist (Supplemental Fig. 3).

### Prior topical application of local anesthetic or transection of the bilateral SLNs completely abolished triggering of the swallowing reflexes by the various stimuli

We applied a local anesthetic (2% lidocaine) into the swallowing-related regions to confirm that triggering of the swallowing reflexes by the various stimuli was caused by excitation of the afferent nerves that carry sensory information from these regions to the sCPG located in the brainstem. Prior (10 min) topical application of lidocaine completely prevented triggering of the swallowing reflexes with various stimuli applied to the SLN-innervated swallowing-related regions (Supplemental Fig. 4).

We also transected the bilateral SLNs to confirm that triggering of the swallowing reflexes by the various stimuli involved the SLN-afferents but not the spinal nerve afferents. In all previous experimental studies (except during recording of the on-site temperature), we recorded the swallowing reflexes with intact bilateral SLNs, but with transection of the other nerves (bilateral IX-ph, X-ph, and lingual branches of the glossopharyngeal [IX-li] nerves, and the recurrent laryngeal nerves [RLN]) that may carry sensory information to the sCPG to trigger the swallowing reflexes. In the present experiments, the bilateral SLNs were also transected. As observed for prior topical application of a local anesthetic, no swallowing reflexes were triggered by various stimuli after transection of the SLNs (Supplemental Fig. 4).

## Discussion

Our findings of TRPA1 expression on thin nerve fibers and fibroblast-like cells located in the subepithelial areas of the SLN-innervated swallowing-related regions are supported by previous findings in human biopsy tissues taken from the pharyngeal and laryngeal regions^18^. In the NPJc, TRPA1s were predominantly expressed on small- to medium-sized SLN-afferent neurons of both myelinated (likely Aδ-type) and unmyelinated (likely C-type) neurons. A small number of large-sized (likely Aβ-type) neurons also expressed TRPA1s. These findings are in agreement with a study that retrogradely traced afferent neurons from the vagus nerve^17^.

Chemical activation of TRPA1s facilitated triggering of the swallowing reflex. Direct activation of TRPA1s located on nerve fibers by chemical agonists and resulting nerve excitation may activate the sCPG in the brainstem to trigger the swallowing reflex. TRPA1s expressed on fibroblasts-like cells may also contribute to triggering of the swallowing reflex. Expression of TRPA1s on non-neuronal cells (e.g., human lung fibroblast cells and odontoblasts) was reported^33–38^. Activation of TRPA1s on fibroblasts by chemical agonists can also cause release of various neurostimulatory mediators including substance P^39^ and adenosine triphosphate^34^, which can act on their respective receptors on nerve fibers and indirectly excite sensory nerves to trigger swallowing reflexes.

The laryngopharyngeal and associated laryngeal regions are richly innervated^40,41^. Previous studies, including those from our group, have reported that chemical stimulation of these regions can modulate SLN activity and trigger swallowing reflexes, which suggests an important role for chemosensors^4,42–46^. In support, several studies including randomized clinical trials in patients with dysphagia have reported improvements in the efficacy, safety, and physiology of swallowing by chemical stimulation in the peripheral swallowing-related regions^4,24,47–53^. Furthermore, a clinical trial of patients with dysphagia reported reduced penetration of bolus particles in the airway when TRPA1 agonists (cinnamaldehyde and zinc) were added to the bolus^24^. Other clinical studies reported facilitation of swallowing function by piperine (a dual activator of TRPA1s and TRPV1s) in dysphagia patients^51,54^. Therefore, targeting TRPA1s in the peripheral swallowing-related regions may be a promising pharmacological treatment strategy for the management of oropharyngeal dysphagia. Recently, pharmacological therapies using TRP channel agonists have been recommended in guideline for the treatment of neurogenic dysphagia as a supplement to behavioral swallowing interventions particularly in patients with a delayed swallow response^55,56^.

In the present study, we also investigated whether TRPA1s in the SLN-innervated regions were activated by cold stimuli to trigger the swallowing reflex. We stimulated these regions with cold saline or AITC and compared the number and intervals of triggered reflexes with those triggered by the same solutions at room temperature. Delivery of saline (at both cold and room temperatures) as a single bolus triggered only one or two swallowing reflexes immediately following delivery, which may be attributed to mechanical stimuli exerted by solution delivery. By contrast, delivery of AITC (at both cold and room temperatures) as a single bolus dose-dependently triggered swallowing reflexes up to 2.5 mM AITC. The initial one or two reflexes observed immediately after AITC delivery may be attributed to mechanical stimuli exerted by the solution delivery. Nevertheless, the later reflexes are likely related to the chemical activation of TRPA1s by AITC. Interestingly, there were no differences in the number or intervals of AITC-induced reflexes between the cold and room temperature solutions, which suggests that application of the cold stimulus to the swallowing related-regions had no additional effect on triggering of the swallowing reflex. The delivery of cold (4 °C) solutions as a single bolus only very briefly reduced the on-site temperature < 17 °C (the temperature at which TRPA1s can be activated), which may explain the lack of effect on triggering of the swallowing reflexes.

To achieve a relatively longer reduction in on-site temperature < 17 °C, cold solutions (4 °C) were delivered continuously for 4 s. However, this increased the time for mechanical stimulation caused by solution delivery, with a resulting increase in the number of triggered reflexes immediately following onset of solution delivery compared with solution delivery as a single bolus; this was particularly evident in the saline group.

The frequency of reflexes/second following cold AITC delivery was significantly reduced during the time period when the on-site temperature was ≤ 17 °C compared with that when the temperature was ≥ 17 °C. However, there was no difference in the frequency of triggered reflexes/second between delivery of cold and room temperature saline, despite the marked difference in on-site temperature. This may relate to the initial mechanical stimuli exerted in the regions by the continuous solution delivery. Thus, these mechanical stimuli may activate the sCPG before starting the action of the cold temperature, resulting in triggering of a similar number of reflexes between delivery of cold and room temperature saline.

We observed that the triggering of chemical stimuli-induced swallowing reflexes was reduced rather than facilitated when the on-site temperature was maintained at levels for a relatively long time at which TRPA1s can be activated. The findings indicate development of cold anesthesia in the regions following prolonged application of cold stimuli. Collectively, our data suggest that TRPA1s in SLN-innervated regions may not function as cold sensors to trigger the swallowing reflex, but rather that a prolonged reduction in on-site temperature, to levels at which TRPA1s can be activated, induces local cold anesthesia.

Previous studies examining the effect of cold stimuli on the swallowing responses have also shown variable and inconclusive results^25–32^. In the majority of those studies, the cold stimulus was induced by touching the mucosa of a swallowing-related region with a pre-cooled metal probe, which results in a combination of cold thermal and mechanical stimuli^25–29^. Additionally, changes in on-site temperature after the stimuli were not recorded. Indeed, a reduction in on-site temperature by touching of a pre-cooled probe can be rapidly reversed because of the high vascularity of the mucosa. Furthermore, the temperature of the pre-cooled probe may increase before touching the mucosa because of exposure to room temperature and the warm oral cavity/throat regions^57^. Finally, mechanical stimuli exerted by touching the mucosa can facilitate the swallowing response^29^. Our experiments revealed that the on-site temperature rapidly increased toward baseline levels after delivery of the cold solutions as a single bolus in the swallowing-related regions, with this very brief cold stimulus having no major effect on triggering of the swallowing reflex. By contrast, a prolong reduction in the on-site temperature by constant application of cold solutions reduced/prevented triggering of the swallowing reflex because of development of cold anesthesia.

We also examined the role of TRPA1s in mechanical-stimuli induced swallowing reflex. Prior topical application of a TRPA1 antagonist at a concentration (2 mM) that significantly reduced the number of AITC-induced swallowing reflexes, or at a concentration more than twice as high (5 mM), did not change the threshold of the punctate mechanical-stimuli induced swallowing reflex. Additionally, there was no effect of the TRPA1 antagonist on triggering of the swallowing reflexes by continuous low-force mechanical pressure stimuli on a vestibular fold. Furthermore, the antagonist had no effect on triggering of the swallowing reflexes by continuous saline delivery for 4 s that exerted mechanical stimuli on the delivery site. These findings suggest that TRPA1s present in the SLN-innervated regions may not act as low-threshold mechanoreceptors to trigger swallowing reflexes. This is supported by our observation of no TRPA1-IR on the sensory corpuscle-like nerve structures/thick nerve fibers present in the SLN-innervated regions, which can be activated by low-force mechanical stimuli. Additionally, TRPA1-IR was observed on a very small percentage of large-sized SLN-afferent neurons in the NPJc. However, the thin nerve fibers present in the SLN-innervated regions and the small- to medium-sized neurons in the NPJc, which can be sensitive to high-force mechanical stimuli, showed robust TRPA1-IR. Interestingly, there was no effect of TRPA1 blockade on triggering of the swallowing reflex by continuous high-force mechanical pressure stimuli, despite using a higher antagonist concentration than that which reduced triggering of the AITC-induced swallowing reflexes. These findings suggest that chemical stimuli, but not mechanical stimuli, can activate TRPA1s present in small- to medium-sized SLN-afferent neurons.

Collectively, our findings indicate that TRPA1s present in the SLN-innervated regions do not act as mechanosensors to trigger the swallowing reflex. Thus, the molecular mechanisms underlying the mechanical stimuli-induced swallowing reflex remain unclear. Speculatively, other mechanosensitive receptors (e.g., TRPV4s, piezo channels, and epithelial sodium channels) may be involved. Indeed, epithelial sodium channels were recently reported to play a role in initiation of low-force punctate mechanical stimuli-induced swallowing reflexes^58^. Further studies are required to fully elucidate the transduction mechanism of the mechanical stimuli-induced swallowing reflex.

We observed two-third reduction of the number of AITC-induced swallowing reflexes following topical application of the TRPA1 antagonist (2 mM), suggesting blocking of the majority of TRPA1 channels by the antagonist. However, the AITC-induced swallowing reflexes were not completely abolished following application of the antagonist, suggesting possible activation of other receptors along with TRPA1s by the AITC.

We have conducted this study in rats but not in mice because in our experience, conducting swallowing related research in mice is difficult because of their size. In our experiences, surgical preparations, electrophysiological recordings, instrumentations are difficult in mice. Many previous swallowing related researches in animals (including our groups) have been conducted in rats. Conducting swallowing research in rats has an advantage of relating the findings of research with previously published findings. However, a limitation of our study is that we have not used TRPA1 knockout rats. Recent progress in developing knockout animals enables to develop knockout rats although making knockout rats may be expensive, time consuming, and needs validation.

In conclusion, our findings suggest that TRPA1s present in the swallowing-related regions act as chemosensors, but not as cold sensors or mechanosensors, to trigger the swallowing reflex. This chemosensor function of TRPA1s may provide an important clinical target for development of pharmacological therapeutics for management of oropharyngeal dysphagia.

## Methods

### Animals and ethical approval

Seventy-four male Sprague–Dawley rats weighing approximately 200–350 g were used in this study (immunohistochemistry, *n* = 10; swallowing reflex, *n* = 57; recording of on-site temperature, *n* = 7). Animals were housed in a room with a 12-h light/12-h dark cycle. Food and water were provided ad libitum. The Intramural Animal Care and Veterinary Science Committee of Matsumoto Dental University approved the protocols (Ref. No. 277, 8 March 2018 and Ref. No. 394, 26 January 2021) and all animals received humane care in accordance with the ARRIVE (Animal Research: Reporting of In Vivo Experiments) guidelines developed by the National Centre for the Replacement, Refinement, and Reduction of Animals in Research. All methods were carried out in accordance with relevant guidelines and regulations. Every effort was made to minimize animal suffering and to reduce the number of animals used.

### Immunohistochemistry

For immunohistochemistry of the SLN-innervated swallowing-related regions, rats were deeply anesthetized and perfused with saline followed by 4% paraformaldehyde. The laryngopharyngeal and associated laryngeal regions were removed and immersed in the same fixative for 24 h at 4 °C. Sections were cut in the sagittal plane (thickness, 50 μm) using a cryostat. Sections were incubated with rabbit polyclonal anti-TRPA1 (1:100; Cat# ACC-037; RRID# AB_2040232; Alomone Labs, Jerusalem, Israel) and mouse monoclonal anti-PGP 9.5 (1:200; Cat# ab8189; RRID# AB_306343; Abcam, Cambridge, UK) antibodies overnight at room temperature, followed by addition of appropriate secondary antibodies (Alexa Fluor 488; Cat# A-11029; RRID#AB_2534088; and Alexa Fluor 594; Cat# A-11037; RRID# AB_2534095; Thermo Fisher Scientific, Waltham, MA) for 2 h at room temperature. Sections were then coverslipped using aqueous mounting medium (PermaFluor; Thermo Fisher Scientific) and examined by fluorescence microscopy (BZ-X700; Keyence Corp., Osaka, Japan).

For NPJc immunohistochemistry, we retrogradely traced the SLN-afferent neurons in the NPJc using the retrograde tracer FG^45,46^. Under sodium pentobarbital anesthesia (50 mg/kg, administered intraperitoneally), the SLN on the right side was isolated after a midline incision in the ventral surface of the neck. The SLN was cut near the trachea and the cut end was inserted into a tube filled with 4% FG. After recovery for 5–7 days, rats were deeply anesthetized and perfused with saline followed by 4% paraformaldehyde^45,46^. The NPJcs were carefully removed and fixed with the same fixative. After preparation, the NPJcs were sectioned (thickness, 16 μm), incubated with rabbit polyclonal anti-TRPA1 (1:2000; Cat# ab58844; RRID# AB_945957; Abcam, Cambridge, UK) and mouse monoclonal anti-NF-200 antibody (1:2000; Cat# N0142; RRID# AB_477257; Sigma-Aldrich, St. Louis, MO) overnight at room temperature, then incubated with appropriate secondary antibodies (Alexa Fluor 488 and 594). The sections were coverslipped and examined using fluorescence microscopy, as described above. IR cells in the region of interest were counted using ImageJ software (National Institutes of Health, Bethesda, MD). Cell counts were performed in the sections showing the highest number of TRPA1-IR cells. Three sections were used from each rat (one section/ganglion). The cell body area of neurons expressing TRPA1 and FG was measured using ImageJ software. A cell area > 1200 *μ*m^2^ was considered large, that 600–1200 *μ*m^2^ was considered medium, and that < 600 *μ*m^2^ was considered small (Table 1)^59,60^.

Sections of the trigeminal ganglions (TG) were used as positive controls for the anti-TRPA1 antibodies used in this study (Supplemental Fig. 1). Previous studies have reported the localization of TRPA1s in TG neurons^61,62^. Universal negative control reagent (Cat# ADI-950-231-0025; Enzo Life Sciences, Inc., Farmingdale, NY)^63^ was used as a primary antibody negative control (Supplemental Fig. 1). Previous studies have used the same anti-TRPA1 antibody to detect TRPA1s^61,62,64,65^.

### Surgical preparation for electrophysiological studies

Rats were anesthetized with urethane (1.0–1.5 g/kg, administered intraperitoneally) and then placed in the supine position^45,46^. The level of anesthesia was monitored during the experiment and supplementary urethane doses were administered if required. A midline incision was made in the ventral surface of the neck and the trachea was isolated from the surrounding tissues. A cannula was inserted into the trachea toward the lungs to maintain respiration. The head of the rat was raised using a pillow made of cotton rolls. A small portion of the trachea (ventral portion only), just below the cricoid cartilage, was surgically removed to create a window to enable stimulating solution delivery^45,46^. This window also reduced the pressure produced in the laryngopharyngeal and associated laryngeal regions during stimulating solution delivery^45,46^.

Because the SLNs were the focus of this study, we transected the other nerves involved in triggering of the swallowing reflex. Specifically, the IX-ph, X-ph, and IX-li nerves, and the RLN of the vagus nerve, were transected bilaterally prior to recording the swallowing reflexes^45,46^. The IX-ph, X-ph, and IX-li branches were exposed by retraction of the posterior belly of the digastric muscles and the horn of the hyoid bone. The RLNs were exposed from either side of the trachea. These procedures provided a condition whereby the bilateral SLNs were intact, while the bilateral RLN, IX-ph, X-ph, and IX-li nerves were transected, during recording of the swallowing reflex following delivery of the different stimulating solutions.

### Recording of the swallowing reflex

During triggering of the swallowing reflex, many infra- and supra-hyoid muscles are activated, including the mylohyoid muscle running from the mandible to the hyoid bone. We identified and counted the number of triggered swallowing reflexes using high amplitude electromyogram (EMG) activity in the mylohyoid muscle and the associated laryngeal movements during triggering of the swallowing reflex^45,46^. Each event that contained high amplitude firing in the EMG signal corresponded with one swallowing reflex^45,46^. Using EMG-signal from the muscles activated during triggering of swallowing reflex is an established method to identify/detect swallowing in scientific studies^1,2,6,8,55,66,67^. Swallowing reflexes were identified by two examiners. The EMG-signal was connected with a speaker, so that the triggering of the swallowing reflexes could be easily understandable by the examiners. Bipolar urethane-coated stainless-steel fine wire electrodes (Unique Medical Co., Ltd., Tokyo, Japan) were implanted into the mylohyoid muscle to record EMG activity during swallowing. EMG signals were amplified and digitized (Power 1401 data acquisition system; Cambridge Electronic Design Ltd., Cambridge, UK), and then stored for later analysis.

### Stimulating solutions

The stimulating solutions were saline (0.9% NaCl, Otsuka Pharmaceutical Co. Ltd. Tokyo, Japan) and AITC (Wako Pure Chemical Industries Ltd. Osaka, Japan; 0.25 mM, 0.5 mM, 1 mM, 2.5 mM, 5 mM, and 10 mM diluted in saline). The pH and osmolarity of the different concentrations of AITC were similar or very near to the saline (Supplemental Table 1). The pH of the solutions was measured using a pH meter (HM-30S, TOA Electronic Ltd. Tokyo, Japan) and the osmolarity of the solutions was measured using a micro-osmometer (Model-210, Fiske Associates, Massachusetts, USA). In pilot studies, the concentrations of AITC were determined as those that evoked a considerable number of swallowing reflexes. The stimulating solutions were delivered topically into the laryngopharyngeal and associated laryngeal regions using a syringe and a 21-gauge needle with a blunted tip. Before solution delivery, any mucous present in the target regions was removed via aspiration. During stimulating solution delivery, the blunted needle tip was placed into the window (surgically prepared just below the cricoid cartilage) and directed toward the laryngopharyngeal and associated laryngeal regions. We then recorded the swallowing reflexes for 20 s following stimulating solution delivery. The time interval between delivery of the various stimulating solutions was 2–3 min. During this time period, the delivered solutions were aspirated out using saline, which was repeated several times to thoroughly wash the region. Pointed pieces of tissue paper were inserted through the window to absorb the remaining saline.

### Temperature and volume of the solutions

The temperature of the stimulating solutions varied depending on the experiments. Different concentrations of room temperature (22–24 °C) AITC were used for experiments testing the concentration-dependent effects of the chemical TRPA1 agonist on triggering of the swallowing reflex. Cold saline (4 °C) was used to examine the effect of putative TRPA1 activation by cold stimuli on triggering of the swallowing reflex. Different concentrations of cold AITC (4 °C) were used to examine the effect of putative TRPA1 activation by dual cold and chemical stimuli on triggering of the swallowing reflex. During these experiments, saline and the various AITC solutions were kept in containers in a pot of ice before delivery. The delivery syringe/needle was also kept in the pot of ice before use to help keep the solutions cold. Generally, we delivered 50 *μ*L of a stimulating solution as a single bolus (in 1 s) toward the laryngopharyngeal and associated laryngeal regions and recorded the triggered swallowing reflexes for 20 s, unless stated otherwise. In one experiment, we delivered 250 *μ*L of room temperature (22–24 °C) or cold temperature (4 °C) AITC (2.5 mM) in 4 s. In another experiment, iced AITC/saline (50 *μ*L solutions) were used to examine the effect of putative TRPA1 activation by low temperatures on triggering of the swallowing reflex.

### Measuring the changes in on-site temperature following solution delivery

The temperature change at the site of solution delivery was measured using ceramic-coated fine surface temperature sensors with a 0.20-mm tip diameter (ST-55 K-CB; RKC instrument Inc., Tokyo, Japan) connected to a thermocouple thermometer (AG 500; Code: K-35; RKC instrument Inc.). These sensors can detect temperature changes with a sampling time of 0.25 s and an accuracy of ± 0.5%. The thin tips of the temperature sensors allowed their placement onto the mucosa of the target regions for measurement of temperature changes at the solution delivery site. Because the shaft of the sensors was flexible, their position could be adjusted and maintained by attachment to the skin with adhesive tape. Note that placing of the sensor tips onto the mucosa of the target site triggered swallowing reflexes due to mechanical stimulation. This phenomenon will introduce errors in the number of triggered swallowing reflexes if the on-site temperature and the number of swallowing reflexes triggered by the stimulating solutions are assessed simultaneously in the same rats. To avoid this problem, we recorded the on-site temperature changes following delivery of different stimulating solutions in a separate cohort of seven rats not used for counting the swallowing reflexes. In these animals, all nerves that may carry sensory information from the target regions (bilateral SLN, IX-ph, IX-li, and RLN nerves) were transected to prevent triggering of the swallowing reflexes during recording of the on-site temperature. Avoiding triggering of the swallowing reflexes during recording of the on-site temperature also allowed us to maintain attachment of the sensor tip to the mucosa of the stimulating site.

### TRPA1 antagonist

HC-030031 (Wako Pure Chemical Industries Ltd., Osaka, Japan) was used as a TRPA1 antagonist. HC-030031 is a well-known TRPA1 antagonist. The efficacy of HC-030031 in blocking TRPA1s was previously valiadted^68–70^. HC-030031 was dissolved in a small amount of DMSO (1%; Sigma-Aldrich, St. Louis, MO) and Tween 80 (1%; Sigma-Aldrich) and was diluted with saline. The corresponding DMSO/Tween 80/saline solution was used as the vehicle. Pilot experiments were performed to determine the lowest effective concentrations of HC-030031 that attenuated the number of swallowing reflexes to ≤ 50% of that triggered by the 2.5 mM AITC. The lowest effective concentrations of HC-030031 that attenuated the number of AITC-induced swallowing reflexes to ≤ 50% was 2 mM. Therefore, we used 2 mM HC-030031 in the remaining experiments where the effect of TRPA1 antagonist on the AITC-induced swallowing reflexes was tested. We used 2 mM and 5 mM HC-030031 in the experiments where the effect of TRPA1 antagonist on the mechanical stimuli-induced swallowing reflexes was tested. The number of AITC-induced/mechanical stimuli-induced swallowing reflexes was counted 10 min following application of the TRPA1 antagonist or vehicle.

### Mechanical stimuli-induced swallowing reflex

We measured the threshold of the punctate mechanical stimuli required to trigger a swallowing reflex from the right and left vestibular folds and from the area between these folds using von-Frey filaments (Aesthesio Tactile Sensory Evaluator Kit, San Jose, CA). These areas of the SLN-innervated regions were chosen because they were comparatively easier to stimulate with the von-Frey filaments than other areas under the supine positioning of the animals. Additionally, the vestibular folds were highly sensitive to mechanical stimuli for triggering a swallowing reflex. A punctate mechanical stimulus triggers a swallowing reflex in an all-or-none manner at the threshold force^5,58^. We also used a custom-made filament (made of thin nylon monofilament) with a force of 0.004 g (calibrated using a digital analytical balance).

To expose the vestibular folds and the area between the folds, a midline incision was made on the ventral surface of the larynx up to the base of the epiglottis. The flaps on both sides of the incision were then retracted by suturing them with threads. The threshold of the punctate mechanical stimuli-induced swallowing reflex was defined as the force (g) that triggered a swallowing reflex using a series of von Frey filaments^58^. To examine the contribution of TRPA1s on the mechanical stimuli-induced swallowing reflex, the activation threshold was measured before and at 10 min following delivery of the TRPA1 antagonist. The antagonist was used in two concentrations—2 mM (the concentration that significantly reduced the number of AITC-induced swallowing reflexes) and 5 mM (more than double the concentration that significantly reduced the number of AITC-induced swallowing reflexes). To assess the contribution of TRPA1s in triggering of the swallowing reflex by continuous low-force mechanical pressure stimuli, the von-Frey filament force at the threshold level was applied to the right/left vestibular folds for 20 s and the number of triggered reflexes was counted before and after TRPA1 antagonist application (5 mM). Additionally, to assess the contribution of TRPA1s in triggering of the swallowing reflex by continuous high-force mechanical pressure stimuli, 1 g of von-Frey filament force was applied to the right/left vestibular folds for 20 s and the number of triggered swallowing reflexes was counted before and after application of the TRPA1 antagonist (5 mM). To assess the contribution of TRPA1s in triggering of the swallowing reflex by delivery of saline continuously for 4 s, the numbers and intervals of triggered swallowing reflexes calculated before and at 10 min following delivery of the TRPA1 antagonist (5 mM).

### Data and statistical analysis

We counted the number of triggered swallowing reflexes for 20 s following application of the stimulating solutions, unless stated otherwise. We also calculated the average interval between the swallowing reflexes from the reflexes evoked within the 10-s time period following the onset of stimulating solution delivery^45,46^. We chose 10-s time period for calculating the interval, because, AITC-induced shortening of interval between the swallowing reflexes was most prominent within this time period. The time interval between the start of high amplitude EMG activity for one swallowing reflex and the start of high amplitude EMG activity for the subsequent swallowing reflex was used as the interval between the respective swallowing reflexes^45,46^. The changes in on-site temperature following delivery of the solutions were averaged from seven rats.

Tests for normality and equal variances were initially performed to decide whether to run parametric or non-parametric statistical tests. The number and intervals of the triggered swallowing reflexes with the different AITC concentrations were compared using one-way repeated measures analysis of variance (ANOVA) followed by Tukey’s test. The number and intervals of the triggered swallowing reflexes with and without prior application of the TRPA1 antagonist/vehicle were compared using Kruskal–Wallis one-way ANOVA on ranks followed by Tukey’s test. The number and intervals of the triggered swallowing reflexes using cold and room temperature solutions (saline and different AITC concentrations) and using cold and iced solutions were compared using a paired *t*-test or Wilcoxon’s signed rank test. The frequency of the triggered swallowing reflexes/second following continuous delivery of saline at cold and room temperatures was compared using Wilcoxon’s signed rank test. The frequency of the triggered swallowing reflexes/second between the different time periods following continuous delivery of AITC was compared using a one-way repeated measures ANOVA followed by Tukey’s test. The thresholds for the punctate mechanical stimuli-induced swallowing reflex with and without prior application of the TRPA1 antagonist/vehicle were compared using the Friedman repeated measures ANOVA on ranks. The number of triggered swallowing reflexes by continuous mechanical pressure stimuli between with and without prior application of the TRPA1 antagonist was compared using a paired *t*-test or Wilcoxon’s signed rank test. The number and intervals of the triggered swallowing reflexes by continuous delivery of saline for 4 s between with and without prior application of the TRPA1 antagonist were compared using a paired *t*-test or Wilcoxon’s signed rank test. Differences were considered significant at *P* < 0.05. All data are presented as the mean ± standard error of the mean (SEM). Statistical analyses were performed using Sigmaplot software (Sigmaplot 14.0; Systat Software Inc., San Jose, CA). The column graphs with individual data points were created using graphing software (GraphPad Prism Software v9.0; GraphPad; San Diego, CA).

## Supplementary Information

Supplementary Figure legends.

Supplementary Figure 1.

Supplementary Figure 2.

Supplementary Figure 3.

Supplementary Figure 4.

Supplementary Table 1.

## Data Availability

Data, analytic methods, and study materials will be made available to other researchers from the authors on reasonable request.
