# Supplementary material for: TRPA1s act as chemosensors but not as cold sensors or mechanosensors to trigger the swallowing reflex in rats
Source: Sci Rep. 2022 Mar 2;12:3431. doi: 10.1038/s41598-022-07400-3 (PMC8891345; doi:10.1038/s41598-022-07400-3)
Supplement: Supplementary file 1 — Supplementary Figure legends. [file 41598_2022_7400_MOESM1_ESM.docx]

**TRPA1s act as chemosensors but not as cold sensors or mechanosensors to trigger the swallowing reflex in rats**

**Mohammad Zakir Hossain, Hiroshi Ando, Shumpei Unno & Junichi Kitagawa**

**Supplemental figure legends**

**Supplemental figure 1**. **Positive and negative controls for TRPA1 antibodies.** (*A*) Representative photomicrographs of a section of rat TG used as a positive control for the rabbit polyclonal anti-TRPA1 (1:2000; Cat# ab58844; Abcam, Cambridge, UK) and mouse monoclonal anti-NF-200 antibodies. White arrows indicate examples of cells positive for TRPA1 and NF-200. White arrowheads indicate examples of cells positive for TRPA1, but negative for NF-200. Scale bars = 100 *μ*m. (*B*) Representative photomicrographs of a section of rat TG used as a positive control for the rabbit polyclonal anti-TRPA1 (1:100; Cat# ACC-03; Alomone Labs, Jerusalem, Israel) and mouse monoclonal anti-NF-200 antibodies. White arrows indicate examples of cells positive for TRPA1 and NF-200. White arrowheads indicate examples of cells positive for TRPA1, but negative for NF-200. Scale bars = 100 *μ*m. (*C*) Representative photomicrographs of a section of the NG used as negative control tissue for the anti-TRPA1 and anti-NF-200 primary antibodies. Universal negative control reagent containing a mixture of purified rabbit, mouse, and goat immunoglobulins was used as the negative control for the primary antibodies. Scale bars = 100 *μ*m. (*D*) Representative photomicrographs of a section taken from the SLN-innervated region used as negative control tissue for the anti-TRPA1 and anti-PGP 9.5 primary antibodies. DAPI was used to visualize the cell nuclei. Universal negative control reagent containing a mixture of purified rabbit, mouse, and goat immunoglobulins was used as the negative control for the primary antibodies. Scale bars = 50 *μ*m.

**Supplemental figure 2. TRPA1 expression is absent on sensory corpuscle-like nerve structures and** **on** **thick nerve fibers in the laryngopharyngeal and associated laryngeal regions.** (*A*) Schematic drawings of the laryngopharyngeal and associated laryngeal regions. Rectangles with arrows and letters show the regions where the photomicrographs were taken. (*B*) Sensory corpuscle-like nerve structures devoid of TRPA1 expression. (*C, D, E*) Thick nerve fibers present in different areas of the swallowing-related regions devoid of TRPA1 expression. Scale bars = 50 *μ*m. The sections were viewed, captured, and examined using a fluorescence microscope.

**Supplemental figure 3**. **Prior topical application of the TRPA1 antagonist had no effect on the triggering of swallowing reflexes induced by delivery of saline continuously for 4 seconds** (*A*) Representative figures of the swallowing reflexes triggered by delivery of saline continuously for 4 seconds with and without prior application of the TRPA1 antagonist. Black arrowhead indicates the onset of stimulating solution delivery. (*B*) Comparison of the numbers of swallowing reflexes triggered by delivery of saline continuously for 4 seconds with and without prior application of the TRPA1 antagonist. (*C*) Comparison of the intervals between the swallowing reflexes triggered by delivery of saline continuously for 4 seconds with and without prior application of the TRPA1 antagonist. n = 5. The number of triggered swallowing reflexes counted for 20 seconds following application of the stimulating solutions and the intervals between the swallowing reflexes calculated from the reflexes evoked within the 10-second time period following the onset of stimulating solution delivery. Data are presented as mean ± SEM. Circles in the column graphs represent individual data points. There were no differences in the number and interval of triggered reflexes between with and without prior application of the TRPA1 antagonist (paired *t*-test or Wilcoxon’s signed rank test).

**Supplemental figure 4**. **Prior topical application of a local anesthetic or transection of the bilateral SLNs completely prevented triggering of the swallowing reflexes by various stimuli.** (*A*) Representative figures of the swallowing reflexes triggered by chemical (topical application of AITC) and mechanical (continuous high-force mechanical-pressure on a vestibular fold) stimuli without prior topical application of lidocaine (2%) or bilateral SLN transection. Black arrowhead indicates the onset of stimulating solution delivery. Blue solid line indicates the duration of continuous mechanical pressure stimuli applied by the von-Frey filaments. (*B*) Representative figures showing no triggered swallowing reflexes when chemical (topical application of AITC) and mechanical (continuous high-force mechanical-pressure on a vestibular fold) stimuli were applied with prior topical application of lidocaine (2%) or bilateral SLN transection. Black arrowhead indicates the onset of stimulating solution delivery. Blue solid line indicates the duration of continuous mechanical pressure stimuli applied by the von-Frey filaments. Four rats (two for lidocaine, two for SLN transection) were used for this experiment. No rats showed any swallowing reflexes triggered by chemical or mechanical stimuli applied in the SLN-innervated swallowing-related regions following topical application of lidocaine or bilateral SLN transection.
