# Supplementary figures and images for: TRPA1s act as chemosensors but not as cold sensors or mechanosensors to trigger the swallowing reflex in rats
Source: Sci Rep. 2022 Mar 2;12:3431. doi: 10.1038/s41598-022-07400-3 (PMC8891345; doi:10.1038/s41598-022-07400-3)

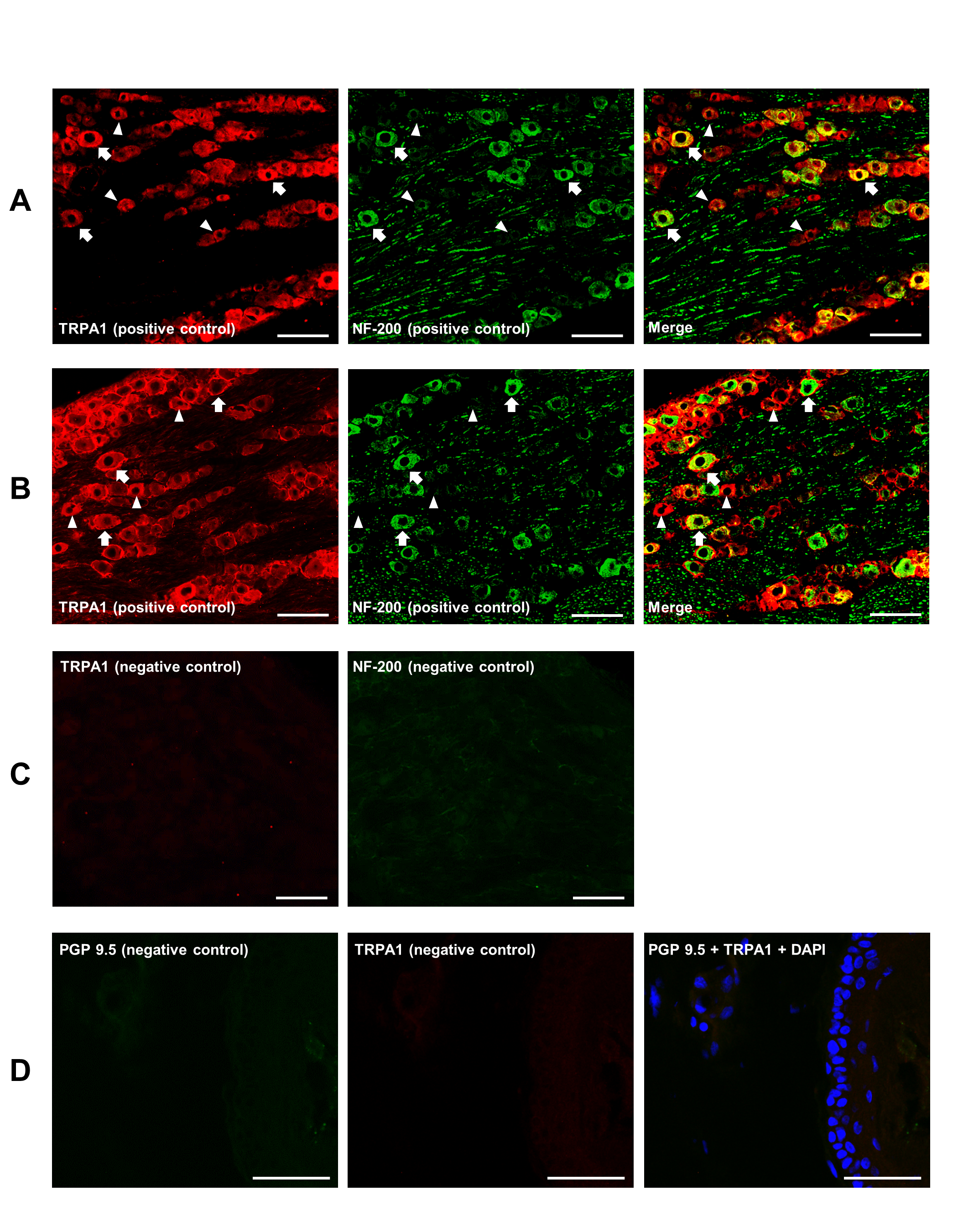

Supplement: Supplementary file 2 — Supplementary Figure 1. [file 41598_2022_7400_MOESM2_ESM.tif]

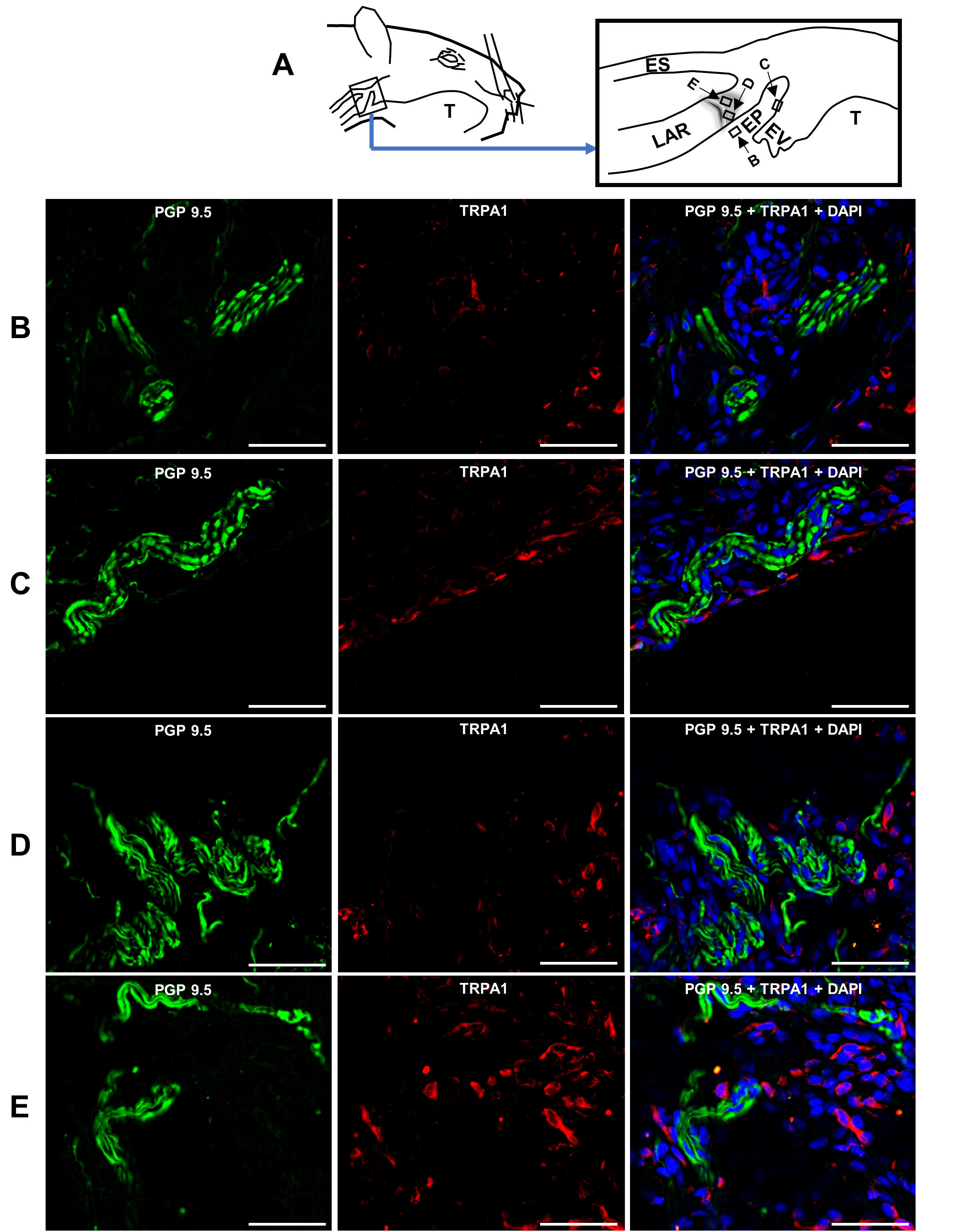

Supplement: Supplementary file 3 — Supplementary Figure 2. [file 41598_2022_7400_MOESM3_ESM.tif]

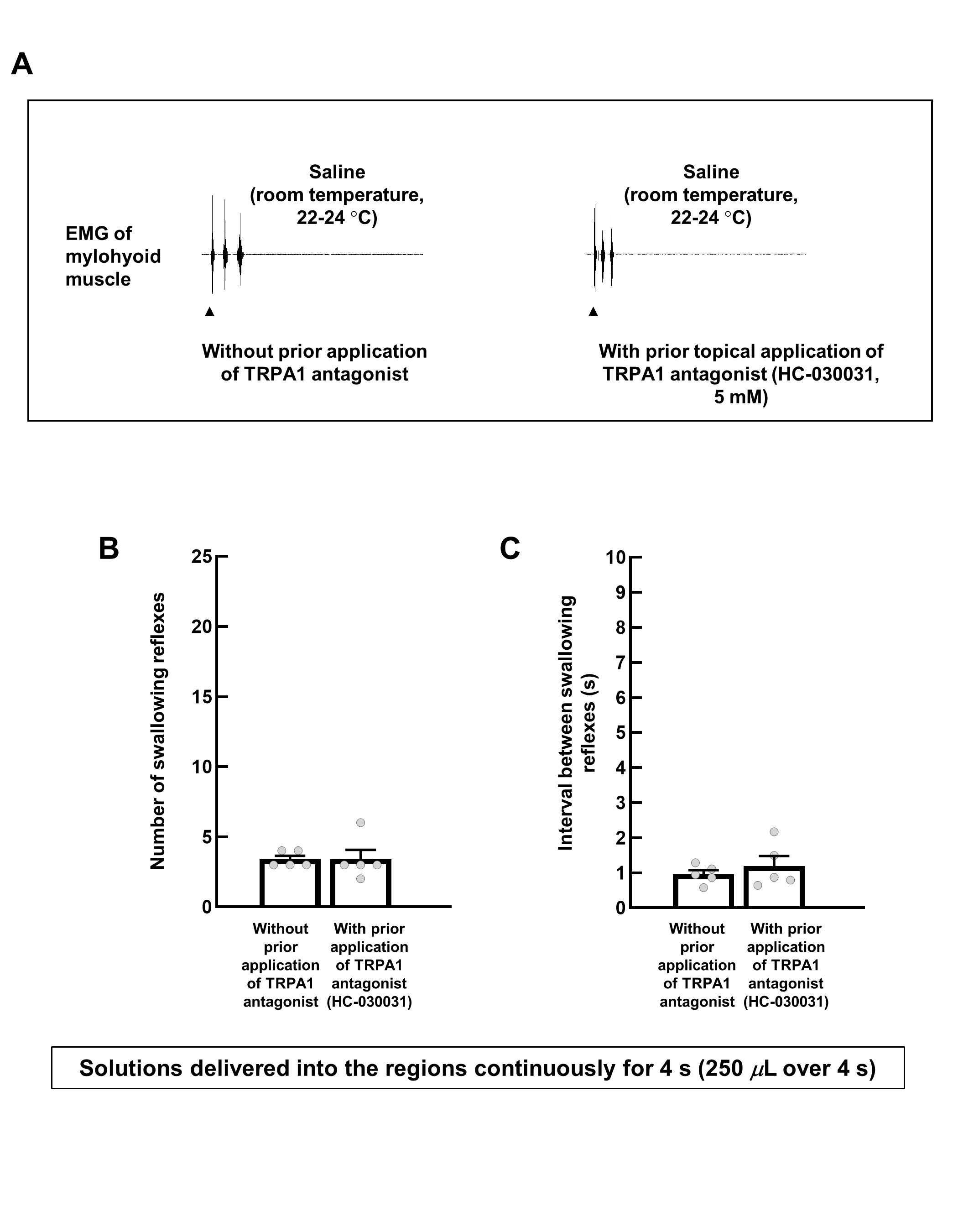

Supplement: Supplementary file 4 — Supplementary Figure 3. [file 41598_2022_7400_MOESM4_ESM.tif]

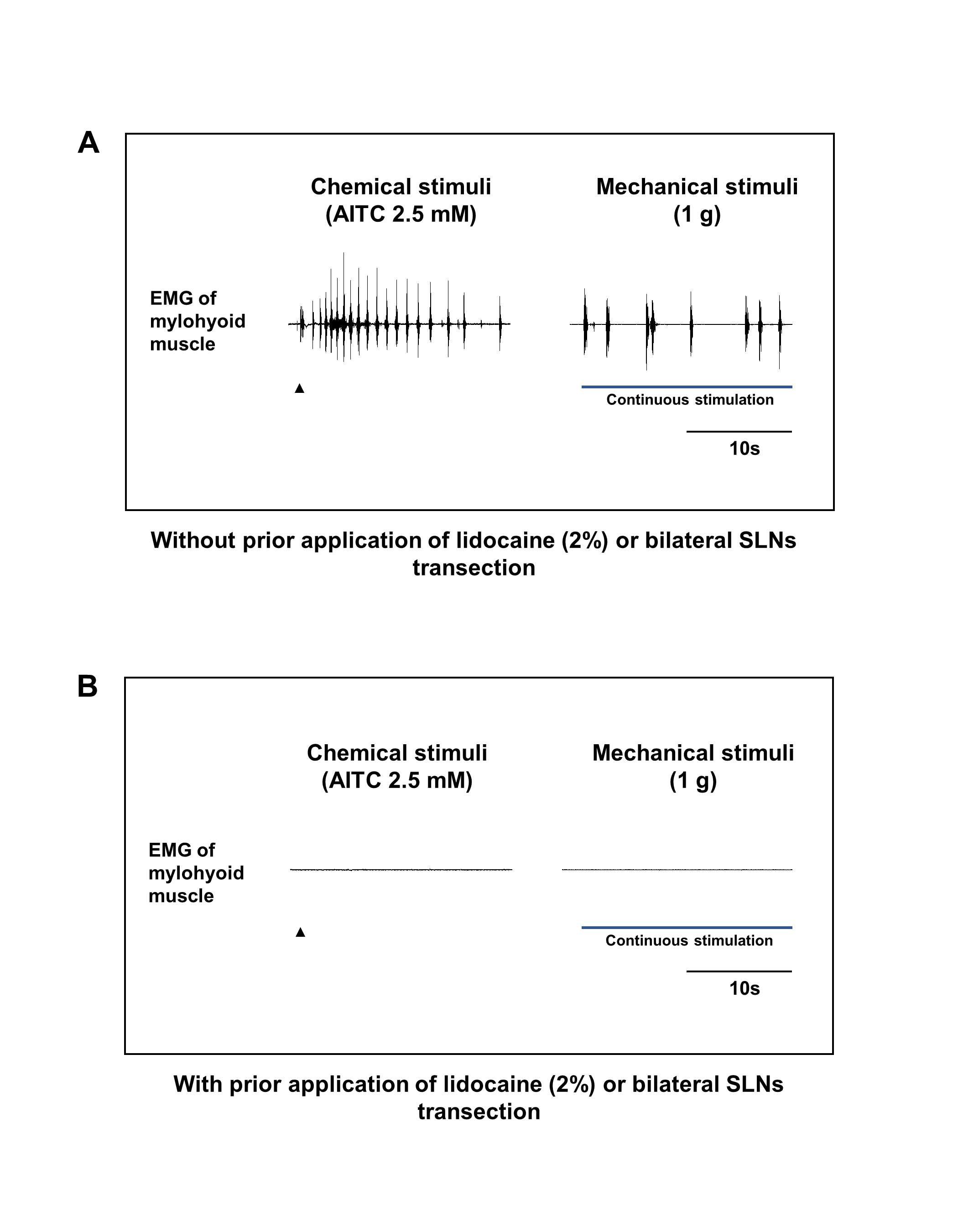

Supplement: Supplementary file 5 — Supplementary Figure 4. [file 41598_2022_7400_MOESM5_ESM.tif]
