# Supplementary material for: TRPA1s act as chemosensors but not as cold sensors or mechanosensors to trigger the swallowing reflex in rats
Source: Sci Rep. 2022 Mar 2;12:3431. doi: 10.1038/s41598-022-07400-3 (PMC8891345; doi:10.1038/s41598-022-07400-3)
Supplement: Supplementary file 6 — Supplementary Table 1. [file 41598_2022_7400_MOESM6_ESM.docx]

**TRPA1s act as chemosensors but not as cold sensors or mechanosensors to trigger the swallowing reflex in rats**

**Mohammad Zakir Hossain, Hiroshi Ando, Shumpei Unno & Junichi Kitagawa**

**Supplemental table 1.** **pH and** **osmolarity of different stimulating solutions**

|  | **Saline** | **AITC**  **0.25 mM** | **AITC**  **0.5 mM** | **AITC**  **1 mM** | **AITC**  **2.5 mM** | **AITC**  **5 mM** | **AITC**  **10 mM** |
| --- | --- | --- | --- | --- | --- | --- | --- |
| **pH** | 5.34  ± 0.01 | 5.32  ± 0.01 | 5.33  ± 0.01 | 5.31  ± 0.01 | 5.33  ± 0.02 | 5.31  ± 0.01 | 5.32  ± 0.02 |
| **Osmolarity (mOsmol/kg)** | 286.6  ± 0.25 | 286.4  ± 0.51 | 286.8  ± 0.49 | 287.4  ± 0.25 | 289.4  ± 0.25 | 290  ± 0.45 | 292.4  ± 0.40 |

Data are presented as mean ± SEM from 5 measurements of each solutions. Note that pH and osmolarity of the stimulating solutions were similar or very near to the saline.
